# The gut metabolite 3-hydroxyphenylacetic acid rejuvenates spermatogenic dysfunction in aged mice through GPX4-mediated ferroptosis

**DOI:** 10.1186/s40168-023-01659-y

**Published:** 2023-09-27

**Authors:** Zirun Jin, Yuzhuo Yang, Yalei Cao, Qi Wen, Yu Xi, Jianxing Cheng, Qiancheng Zhao, Jiaming Weng, Kai Hong, Hui Jiang, Jing Hang, Zhe Zhang

**Affiliations:** 1 Department of Urology, Center for Reproductive Medicine, Peking University Third Hospital, 49 North Garden Road, Haidian District, Beijing, 100191 China; 2 Department of Urology, Peking University First Hospital, Xishiku Road, Xicheng District, Beijing, 100034 China; 3 Institute of Urology, Peking University, Beijing, China; 4 Department of Andrology, Peking University First Hospital, Beijing, China; 5 Department of Obstetrics and Gynecology, Peking University First Hospital, Beijing, China; 6 Department of Obstetrics and Gynecology, State Key Laboratory of Female Fertility Promotion, Peking University Third Hospital, 49 North Garden Road, Haidian District, Beijing, 100191 China; 7 Key Laboratory of Assisted Reproduction, Ministry of Education, Beijing, China; 8 Beijing Key Laboratory of Reproductive Endocrinology and Assisted Reproduction, Beijing, China; 9 National Clinical Research Center for Obstetrics and Gynecology, Beijing, China

**Keywords:** Aging, Spermatogenic dysfunction, Gut microbiota, 3-Hydroxyphenylacetic acid, Ferroptosis

## Abstract

**Background:**

Aging-related fertility decline is a prevalent concern globally. Male reproductive system aging is mainly characterized by a decrease in sperm quality and fertility. While it is known that intestinal physiology changes with age and that microbiota is shaped by physiology, the underlying mechanism of how the microbiota affects male reproductive aging is still largely unexplored.

**Results:**

Here, we utilized fecal microbiota transplantation (FMT) to exchange the fecal microbiota between young and old mice. Cecal shotgun metagenomics and metabolomics were used to identify differences in gut microbiota composition and metabolic regulation during aging. Our results demonstrated that FMT from young to old mice alleviated aging-associated spermatogenic dysfunction through an unexpected mechanism mediated by a gut bacteria-derived metabolite, 3-hydroxyphenylacetic acid (3-HPAA). 3-HPAA treatment resulted in an improvement of spermatogenesis in old mice. RNA sequencing analysis, qRT-PCR and Western blot revealed that 3-HPAA induced an upregulation of GPX4, thereby restraining ferroptosis and restoring spermatogenesis. These findings were further confirmed by in vitro induction of ferroptosis and inhibition of GPX4 expression.

**Conclusions:**

Our results demonstrate that the microbiome-derived metabolite, 3-HPAA, facilitates spermatogenesis of old mice through a ferroptosis-mediated mechanism. Overall, these findings provide a novel mechanism of dysregulated spermatogenesis of old mice, and suggest that 3-HPAA could be a potential therapy for fertility decline of aging males in clinical practice.

Video Abstract

**Supplementary Information:**

The online version contains supplementary material available at 10.1186/s40168-023-01659-y.

## Background

Over the past few decades, advances in medicine and public health policy have led to significant increase in life expectancy worldwide. However, we are now spending more years in poor health [1]. This is primarily due to the effects of aging, which is characterized by a progressive loss of physiological integrity and driven by twelve hallmarks, such as genomic instability, chronic inflammation, and dysbiosis. These hallmarks are known to lead to impaired physiological function and increased susceptibility to death [2, 3]. It is worth noting that aging is also associated with changes in the hypothalamic-pituitary–gonadal axis and a gradual decline in gonadal function. The decline in gonadal function-induced reproductive aging promotes the development and progression of several comorbidities, including chronic vascular and metabolic disorders [4, 5].

In modern society, the aging-related fertility decline has become increasingly prevalent due to the trend of postponing first-time motherhood [6, 7]. Testicular function in men gradually deteriorates with aging, which impairs their fertilizing capacity, overall health status, and quality of life [8]. The aging process of the male reproductive system, characterized by declining levels of sexual hormones, sperm quality, and fertility, may be attributed to mitochondrial dysfunction, increased oxidative stress, and necroptosis [8–11].

Intestinal physiology plays a crucial role in shaping gut microbiota (GM), and its changes with age may contribute to the persistence or outgrowth of certain microbes [12, 13]. The alteration of human GM is associated with changed systemic hormones and spermatogenesis, and nutrients such as vitamins and minerals metabolized by GM are vital to male reproduction via the gut-testis axis [14, 15]. Age-associated microbial dysbiosis could promote intestinal permeability, systemic inflammation, macrophage dysfunction and cancer risk through regulating the host serum metabolome and brain lipid composition [16–19]. Fecal microbiota transplantation (FMT) from aged donor mice affects spatial learning and memory in young recipients via modulation of hippocampal synaptic plasticity- and neurotransmission-related proteins, whereas FMT from young mice to aged mice reverses hallmarks of the aging and selective age-associated behavioral deficits [20–23]. Moreover, accumulating evidence shows that the GM plays an important role in regulating spermatogenesis in testis tissues [24], and that its disturbance is closely associated with impairment of spermatogenesis, sperm motility, and alterations in GM-affected testicular dysfunction, which may occur through disruption of polyamine metabolism [25–27]. However, it remains unclear whether and how GM contributes to spermatogenic dysfunction of male aging mice.

In the present study, we investigated the spermatogenic dysfunction and GM composition of aging mice and examined the cecal metabolome, cecal, plasma, and testicular metabolic patterns after FMT. Additionally, we investigated the potential roles and underlying mechanisms of GM in spermatogenic dysfunction of aging mice.

## Methods

### Mice

Male C57BL/6J mice aged 6 weeks and 18–20 months were housed in individually ventilated cages with three to five mice per cage and maintained on a 12-h light/dark cycle at 23.0 ± 3.0 °C with free access to a standard chow diet (4% fat, 18% protein, 50% carbohydrates, and 5% fiber) and water under specific pathogen-free (SPF) conditions at Peking University Health Science Center. All animal experiments were approved by the Animal Care and Use Committee of Peking University (LA2021371).

### Fecal microbiota transfer

For microbiota transplantation, the mice (15 mice of 6 weeks or 18–20 months) cecum fecal samples (100 mg per mice) were collected under sterile conditions and were resuspended in a concentration of 50 mg/mL in pre-cooled PBS (with 20% glycerin), centrifuged with 1000 rpm for 10 min at 4 °C, then the supernatant were storaged at − 80°C until use [28].

**Fig. 1:**
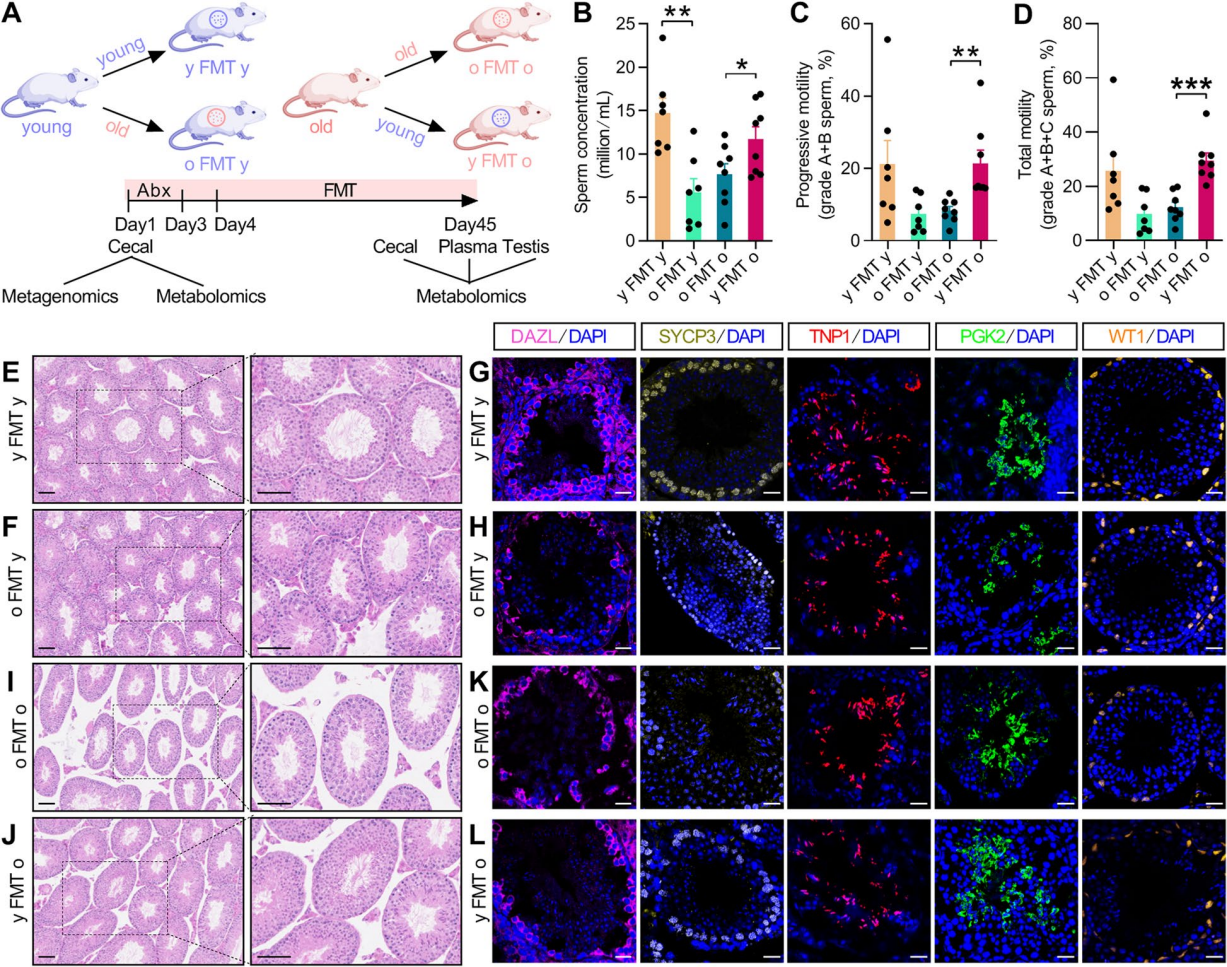
Heterochronic fecal microbiota transfer affects spermatogenesis of young and old mice. **A** The experimental workflow for fecal microbiota transplantation (FMT) and multi-omics examination of young (6-week), old (18–20-month), and FMT mice. For FMT groups, y FMT y and o FMT y (damaged comparison) indicate young mice received young or old donor microbiota, respectively; o FMT o and y FMT o (rescued comparison) indicate old mice received old or young donor microbiota, respectively. **B**–**D **Sperm quality including sperm concentration (**B**), progressive motility (grade A + B sperm) (**C**), and total motility (grade A + B + C sperm) (**D**). **E**–**L** Representative images of H&E staining (**E**, **F**, **I**, **J**) and immunofluorescence staining of testis tissues (**G**, **H**, **K**, **L**) for FMT mice. DAZL (spermatogonia marker), SYCP3 (spermatocyte marker), TNP1 (spermatid marker), PGK2 (spermatozoa marker), and WT1 (Sertoli cell marker) were stained. Scale bar = 100 μm for **E**, **F**, **I**, **J** or 25 μm for **G**, **H**, **K**, **L**. All data are presented as mean ± SEM. *P < 0.05; **P < 0.01; ***P < 0.001. Data are analyzed by two-tailed unpaired Student’s* t* test. *n* = 7–8 mice per group

After 2 weeks of adaptation, the young mice (6 weeks, y) or old mice (18–20 months, o) were randomly assigned into four groups and designated for fecal microbiota transplantation (FMT) interventions (see Fig. 1A). We depleted the host microbiota by prior antibiotic cocktail administration in drinking water (vancomycin 0.5 g/L, ampicillin 1 g/L, kanamycin 1 g/L, and metronidazole, 1 g/L) for 3 days, then the mice were maintained with free access to food and water [28]. The young mice were intragastrically adiministation of fecal microbiota (100 μL for each mice) from young mice (young FMT to young, y FMT y, 7 mice) or old mice (old FMT to young, o FMT y, 7 mice), as well as old mice were intragastrically adiministation of fecal microbiota (100 μL for each mice) from old mice (old FMT to old, o FMT o, 8 mice) or young mice (young FMT to old, y FMT o, 8 mice) at 1-day interval for 6 weeks [21].

### Sperm count and motility assessment

Cauda epididymal sperm of the mice were collected and prepared as described previously [29, 30]. In brief, two caudal epididymis samples were placed into human tubal fluid medium (HTF), then, the cauda epididymis was slightly cut into three pieces and incubated in a 5% CO_2_ incubator at 37 °C for 5 min. Ten microliters sperm suspension was used for assessment of sperm count and motility by a computer-assisted semen analysis (CASA) system (SSA-II, Gold Edition, SuiJia Software, Beijing, China) according to the laboratory manual of the World Health Organization for sperm concentration and sperm motility [31]. The following parameters were evaluated: sperm concentration (10^6^/mL), progressive motility (grade A + B, %), total motility (grade A + B + C, %), straight-line velocity (VSL, μm/s), curve-line velocity (VCL, μm/s), average path velocity (VAP, μm/s), amplitude of lateral head displacement (ALH, μm), and straightness (STR, %). A minimum of 200 sperm were counted for each assay.

### Hematoxylin and eosin (H&E) staining

Under deep anesthesia, the testes of the mice were removed quickly and fixed in testicular tissue fixation buffer (G1121, Servicebio, Wuhan, China) for 24 h. Following dehydration through an ethanol series, the fixed testes were embedded in paraffin and then sectioned. Paraffin Sects. (5-µm-thick) were then stained with H&E as previously described [29]. Histological analysis was performed using digital panoramic scanner (WS-10, WISLEAP, Zhiyue Medical Technology Co., LTD; Jiangsu, China).

### Immunofluorescence staining

The paraffin sections were heated at 95 °C in EDTA antigen repair buffer (ZLI-9071, ZSGB-BIO; Beijing, China) for 30 min and cooled into room temperature. The sections were then blocked in 10% donkey serum (in 0.1 M PBS) with 0.3% Triton X-100 for 1 h at room temperature, and incubated with the primary antibodies in 1% donkey serum (in 0.1 M PBS) at 4 °C overnight which includes rabbit polyclonal anti-DAZL (1:100; ab34139, Abcam, Cambridge, UK), mouse monoclonal anti-SYCP3 (1:100; ab97672, Abcam), rabbit polyclonal anti-TNP1 (1:300; ab73135, Abcam), rabbit polyclonal anti-PGK2 (1:300; D121803, Sangon Biotechnology, Shanghai, China), and rabbit monoclonal anti-WT1 (1:50; ab89901, Abcam), respectively. After washing in PBS for three times, tissues were incubated with the following secondary antibodies at room temperature for 1 h: Cy3-conjugated AffiniPure donkey polyclonal anti-mouse IgG (H + L) and Alexa Fluor 488-conjugated AffiniPure donkey polyclonal anti-rabbit IgG (H + L) (1:500; Jackson ImmunoResearch Laboratories, Philadelphia, PA, USA). The tissues were counterstained with the nuclear marker DAPI (100 ng/mL, Beyotime, Jiangsu, China) carrying blue fluorescence for 10 min at room temperature. After three washes in PBS, the slides were mounted in Gel-Mount medium and observed under a confocal microscope (Zeiss LSM710, Carl Zeiss Microscopy GmbH, Jena, Germany) at excitation wavelengths of 488 nm (green), 555 nm (red), and 405 nm (blue).

### Shotgun metagenomic sequencing and analysis

At the endpoint of the experiments, cecum fecal samples (100 mg per mice) were collected under sterile conditions and were stored at − 80°C. Shotgun metagenomic sequencing and analysis were prepared as previously described and supported by Annoroad Gene Technology Co., Ltd, Beijing, China [32]. Genomic DNA was extracted using a QIAGEN kit and monitored by electrophoresis on a 1% agarose gel. The quality of the DNA samples was further quantified using a Qubit^®^ 2.0 fluorometer (Life Technologies; CA, USA) with an OD value between 1.8 and 2.0. For library construction, a total of 1 μg of DNA per sample was used as input material for the DNA sample preparations. Sequencing libraries were generated using the NEBNext® UltraTM DNA Library Prep Kit for Illumina (NEB, USA) following the manufacturer’s recommendations, and index codes were added to attribute sequences to each sample. Briefly, the DNA sample was fragmented by sonication to a size of 350 bp, and then DNA fragments were end-polished, A-tailed, and ligated with the full-length adaptor for Illumina sequencing with further PCR amplification. Finally, PCR products were purified (AMPure XP system), and libraries were analyzed for size distribution by using an Agilent2100 Bioanalyzer and quantified using real-time PCR. After the index-coded sample clusters were generated on a cBot Cluster Generation System according to the manufacturer’s instructions, the library preparations were sequenced on an Illumina NovaSeq platform, and paired-end reads were generated, with at least 6 Gb reads per sample.

We used Readfq (V8, https://github.com/cjfields/readfq) to acquire the clean data for subsequent analysis. Host sequences were then discarded by mapping the sequences against the reference genome (hg19) using BowTie2.2.4 (http://bowtie-bio.sourceforge.net/bowtie2/index.shtml). We pooled and subjected the remaining set of clean reads to metagenomics by using SOAPdenovo software (V2.04, http://soap.genomics.org.cn/soapdenovo.html). Then, we interrupted the assembled scaftigs from the N connection and left the scaftigs without N. All samples’ clean data were compared to each scaffold by using Bowtie2.2.4 software to acquire the PE reads that were not used. The assembled scaftigs (> 500 bp) were predicted as ORFs by MetaGeneMark (V2.10, http://topaz.gatech.edu/GeneMark/) software, and the length information shorter than 100 nt was filtered. CD-HIT software (V4.5.8, http://www.bioinformatics.org/cd-hit) was adopted for redundancy and to obtain the unique initial gene catalog and ORF prediction. DIAMOND software (V0.9.9, https://github.com/bbuchfink/diamond/) was used to BLAST the unigenes to the sequences of bacteria, fungi, archaea, and viruses, which were all extracted from the NR database (Version: 2018–01-02, https://www.ncbi.nlm.nih.gov/). We used the LCA algorithm, which is applied to the system classification of MEGAN software, to ensure the species annotation information of sequences. We adopted DIAMOND software (V0.9.9) to BLAST unigenes to the functional database. The functional database includes the KEGG database (Version 2018–01-01, http://www.kegg.jp/kegg/), eggNOG database (Version 4.5, http://eggnogdb.embl.de/#/app/home), and CAZy database (Version 201,801, http://www.cazy.org/). For each sequence’s BLAST result, the best BLAST hit was used for subsequent analysis.

### Non-targeted metabolic profiling

The untargeted metabolomics profiling was performed on XploreMET platform (Metabo-Profile, Shanghai, China). Gut digesta, plasma, and testis samples were separately subjected to metabolomics analysis. The samples were prepared as described previously [33]. A time-of-flight mass spectrometry (GC-TOF/MS) system (Pegasus HT, Leco Corp., St. Joseph, MO, USA) with an Agilent 7890B gas chromatography and a Gerstel multipurpose sample MPS2 with dual heads (Gerstel, Muehlheim, Germany). A Rxi-5 ms capillary column (30 m × 250 μm i.d., 0.25-μm film thickness; Restek corporation, Bellefonte, PA, USA) was used for separation. Helium was used as the carrier gas at a constant flow rate of 1.0 mL/min. The temperature of injection and transfer interface were both set to 270 °C. The source temperature was 220 °C. The measurements were made using electron impact ionization (70 eV) in the full scan mode (m/z 50–500). The raw data generated by GC-TOF/MS were processed using ChromaTOF (v4.71, Leco Corp., St. Joseph, MO, USA) for automated baseline denosing and smoothing, peak picking, deconvolution, and peak alignment. Compound identification was performed by comparing both MS similarity and FAMEs retention index distance with the referenced standards in JiaLib database. The self-developed platform iMAP (v1.0, Metabo-Profile, Shanghai, China) was used for subsequent statistical analyses, including PCA, OPLS-DA, univariate analysis, and pathway analysis. Unsupervised principal component analysis (PCA) was performed by using the statistics function prcomp within R (www.r-project.org). The data were unit variance scaled before unsupervised PCA. The hierarchical cluster analysis results of the samples and metabolites were presented as heatmaps with dendrograms, while Pearson correlation coefficients between samples were calculated by the cor function in R and presented as only heatmaps. Significantly regulated metabolites between groups were determined by VIP ≥ 1 and absolute FC (fold change) > 1 and p value < 0.05. VIP values were extracted from the OPLS-DA results, which also contained score plots and permutation plots, and were generated using the R package MetaboAnalystR. The data were log transformed (log2) and mean centered before OPLS-DA. To avoid overfitting, a permutation test (200 permutations) was performed. Identified metabolites were annotated using the KEGG compound database (http://www.kegg.jp/kegg/compound/), and annotated metabolites were then mapped to the KEGG pathway database (http://www.kegg.jp/kegg/pathway.html). Significantly enriched pathways were identified with a hypergeometric test p value for a given list of metabolites.

### Metabolite treatment

3-Hydroxyphenylacetic acid (3-HPAA, HY-W001083, MedChemExpress, Shanghai, China) were dissolved in normal saline (10 mg/mL). Old mice were intragastrically administrated of 3-HPAA at a dose of 25 mg/kg or equal amount of normal saline once per day for 6 weeks [34].

### Targeted metabolic profiling

Plasma samples were vortexed for 10 s, then 50 μL supernatant was transferred to a centrifuge tube, mixed with 250 μL of 20% acetonitrile/methanol, and vortexed for another 3 min. An amount of 0.05 g of the testis tissues samples was mixed with 500 µL of 70% methanol/water. All the above samples were vortexed for 3 min under the condition of 2500 r/min and centrifuged at 12,000 rpm for 10 min at 4°C. Three hundred microliters of supernatant was transferred into a new centrifuge tube and placed at − 20°C for 30 min. Then the supernatant was centrifuged again at 12,000 r/min for 10 min at 4°C and 200 μL of supernatant was transferred for further LC–MS analysis.

Organic acid and its metabolites were detected by MetWare (http://www.metware.cn/) based on the AB Sciex Q-TRAP 6500 LC-MS/MS platform. The analytical conditions were as follows, HPLC: column, ACQUITY HSS T3 (i.d.2.1 × 100 mm, 1.8 μm); solvent system, water 0.05% formic acid (A), acetonitrile with 0.05% formic acid (B); the gradient was started at 5% B (0 min), increased to 95% B (8–9.5 min), finally ramped back to 5%B (9.6–12min); flow rate,0.35mL/min; temperature, 40°C; injection volume: 2 μL. AB 6500 + QTRAP® LC–MS/MS System, equipped with an ESI Turbo Ion-Spray interface, operating in both positive and negative ion modes and controlled by Analyst 1.6 software (AB Sciex). The ESI source operation parameters were as follows: ion source, turbo spray; source temperature 550°C; ion spray voltage (IS) 5500 V (Positive), − 4500 V (Negative); Curtain gas (CUR) were set at 35.0 psi; DP and CE for individual MRM transitions was done with further DP and CE optimization. A specific set of MRM transitions were monitored for each period according to the organic acid eluted within this period.

### Transcriptome profiling

Whole-genome gene expression analysis was performed in testis tissue from vehicle and 3-HPAA mice. Total RNA was extracted from the cryopreserved testes by using TRIzol Reagent (Invitrogen, CA, USA). The cDNA samples were sequenced using the Agilent Bioanalyzer 2100 system (Agilent Technologies, CA, USA). The clustering of the index-coded samples was performed on a cBot cluster generation system using HiSeq PE Cluster Kit v4-cBot-HS (Illumina) according to the manufacturer’s instructions. After cluster generation, the libraries were sequenced on an Illumina platform and 150 bp paired-end reads were generated. The cluster generation and sequencing were performed on Novaseq 6000 S4 platform, using NovaSeq 6000 S4 Reagent kit V1.5.

### RNA extraction and RT-qPCR

Total RNA was extracted from the purified testis tissues with TRIzol reagent (Life Technologies). Reverse transcription and PCR was performed with oligo deoxythymidine (oligo-dT) primers and moloney murine leukemia virus reverse transcriptase (Promega) according to the manufacturer’s protocol. PCR primer sequences are listed in table S1. Quantitative real-time PCR (RT-qPCR) assay was performed with PowerUp™ SYBR™ Green Master Mix (Applied Biosystems, CA, USA) and an QuantStudio 3 Real-Time PCR System instrument (Thermo Scientific, CA, USA). Briefly, 20 μL PCR reaction that includes 1 μL of complementary DNA, 10 μL of PowerUp™ SYBR™ Green Master Mix, and 0.2 μM of each primer was used and adjusted to the final volume with double distilled H_2_O (ddH_2_O), while β-actin in parallel for each run was used as an internal control. The reactions were set up on the basis of the manufacturer’s protocol. PCR conditions were incubation at 50°C for 2 min and 95°C for 2 min followed by 40 cycles of thermal cycling (15 s at 95 °C and 1 min at 60 °C). The relative expression ratio of mRNA was quantified via the 2 (^−ΔΔCt^) method [30].

### Western blotting

The deposit of a piece of testis tissues or GC-2 cells were immediately homogenized in ice-cold RIPA lysis buffer (Beyotime) containing 1 mM phenylmethanesulfonyl fluoride (PMSF). The homogenates were centrifuged at 12,000g for 10 min at 4 °C to yield the total protein extract in the supernatant, and then analyzed by Western blotting according to the methods as described elsewhere [30]. The concentration of protein was measured with a bicinchoninic acid (BCA) assay kit (Pierce/Thermo Scientific), and an equal amount of protein samples (40 μg or 25 μg for testis tissues or GC-2 cell lines, respectively) was denatured and then separated through SDS-PAGE using 10% separating gels and transferred to a PVDF membrane (Bio-Rad, Hercules, CA). The membranes were blocked with 5% nonfat milk in TBST buffer (B1009, Applygen, Applygen Technologies Inc. Beijing, China) for 60 min at room temperature and then incubated with the following primary antibodies at 4 °C overnight: rabbit polyclonal to TfR (Transferrin, 1:1000, ab82411, Abcam, Cambridge, UK); rabbit polyclonal to FTL (Ferritin light chain, 1:1000, ab69090, Abcam); rabbit monoclonal anti-FTH1 (Ferritin heavy chain, 1:1000, 4393S, Cell Signaling Technology); rabbit monoclonal to GPX4 (Glutathione peroxidase 4, 1:1000, ab125066, Abcam); rabbit monoclonal to ACSL4 (Long-chain fatty acid CoA ligase 4, 1:1000, ab155282, Abcam), rabbit monoclonal to NRF2 (Nuclear factor E2-related factor2, 1:1000, A21176, ABclonal), rabbit polyclonal to xCT (Cystine/glutamate transporter; Slc7a11, Solute carrier family 7 member 11, 1:1000, ab37185, Abcam), mouse monoclonal to α-tubulin (1:3000, 3873S, Cell Signaling Technology), and mouse monoclonal to β-actin (1:3000, YM3028, ImmunoWay Biotechnology; SuZhou, JiangSu, China), respectively. The blots were incubated in horseradish peroxidase-conjugated secondary antibody including goat anti-rabbit IgG antibody (1:5000, BF03008, Biodragon Immunotechnologies, Suzhou, Jiangsu, China) or goat anti-mouse IgG antibody (1:5000, BF03001, Biodragon Immunotechnologies). Protein bands were visualized using an enhanced chemiluminescence detection kit (Pierce) followed by using a Tanon 5200 chemiluminescence detection system (Tanon, Shanghai, China). The bands were quantified with a computer-assisted imaging analysis system (Image J, NIH).

### Oxidative stress assessments

We used commercial kits purchased from Beyotime Biotechnology according to the manufacturer’s instructions to assess the oxidative stress in testis tissues of mice or GC-2 cell lines as described before [29]. Total antioxidant capacity (cat# S0119), glutathione peroxidase (GPx) (cat# S0058, total glutathione peroxidase assay kit with NADPH), and superoxide dismutase (SOD) (cat# S0109, total superoxide dismutase assay kit with NBT), GSH and GSSG (reduced glutathione, GSH; oxidized glutathione disulfide, GSSG; cat# S0053, GSH and GSSG assay kit), and the levels of malondialdehyde (MDA) (cat# S0131M, lipid peroxidation MDA assay kit) were measured.

### Cell culture, RSL3, and siRNA treatment

GC-2 spd cell line (CL-0593, Procell; Wuhan, China) was cultured in DMEM supplemented with 10% fetal bovine serum (FBS, Hyclone) at 37°C in a humidified atmosphere containing 5% CO_2_. For the flow cytometry analysis, 3 × 10^5^ cells were seeded into one well of 6-well cell culture plates. For other experiments, 7 × 10^5^ cells were seeded into 6-cm cell culture dish. To induce senescence using oxidative stress (OSIS), cells were treated with 200 μM of hydrogen peroxide (H_2_O_2_, Sigma Aldrich) for 90 min, followed by drug removal and culturing in fresh DMEM supplemented with 10% FBS [35]. For the treatment of 3-HPAA, 3-HPAA (50 μM, dissolved in DMSO), or vehicle (0.1% DMSO) were added into the medium and cultured for 48 h after H_2_O_2_ treatment. For the treatment of RSL3 and Gpx4 siRNA, RSL3 (200 nM, dissolved in DMSO, HY-100218A, MedChemExpress) or Gpx4 siRNA (20 nM in Lipofectamine™ 3000 Transfection Reagent, Thermo Fisher) were added into the medium for 48 h after H_2_O_2_ treatment. And siRNA knockdown was observed 48 h after transfection. The siRNA was purchased from GenePharma (Shanghai, China), and their sequences are showed in Table S2 [36, 37].

### Flow cytometry analysis

To analyze the effects of the indicated treatments on cell survival, cells were stained with Annexin V-FITC and PI Detection Kit (C1062M, Beyotime) and analyzed by flow cytometry. Flow cytometry data were assessed using BD FACSDiva Software v7.0 (Becton–Dickinson, USA) [38].

The intracellular ROS levels were detected using a peroxide-sensitive fluorescent probe (DCFH-DA; Beyotime) according to the instructions of the manufacturer. Briefly, DCFH-DA was diluted to a final concentration of 10 μM for 30 min at 37 °C. Later, the cells were harvested and washed twice with PBS and then subjected to flow cytometry [37].

### Iron staining

2 × 10^5^ GC-2 cells were seeded on glass bottom culture dishes (BS-20-GJM, Life sciences). After the treatment of H_2_O_2_, 3-HPAA or GPX4 siRNA, plates were washed 3 times in HBSS, then cells were stained in 1 μM Ferro orange (F374, dojindo) in HBSS for exactly 30 min at 37°C in a humidified atmosphere containing 5% CO_2_ and 95% air and imaged immediately. Confocal images were acquired with a Zeiss inverted LSM 710 laser scanning confocal microscope (Zeiss) using a 20 × /1.4 DIC immersion objective. Five representative fields were captured for each condition under identical exposure times. Images were obtained with the Cy3 filter (ex 514nm, em 525–596). The images are 512 × 512 pixels [39].

### Statistical analysis

Statistical analyses were performed with GraphPad Prism 9.0 for Windows (GraphPad Software, La Jolla, CA). All quantitative biochemical data and immunofluorescence staining were representative of at least three independent experiments. Two-tailed unpaired Student’s *t* test or Wilcoxon test was used for the comparison of the mean values between two groups. One-way ANOVA with Sidak’s *post hoc* test was used for multiple comparisons. All data were expressed as means ± SEM, and differences with *P* < 0.05 were considered statistically significant. The significant differences between groups were represented as * *P* < 0.05, ** *P* < 0.01, and *** *P* < 0.001.

## Results

As indicated in the experimental workflow (Fig. 1A), we investigated the effect of FMT on spermatogenesis in mice of different ages. We performed the FMT between young (6-week-old) and old (18- to 20-month-old) C57BL/6 mice and collected cecum fecal samples (before and after the transplantation), plasma, and testis tissues (after the transplantation) to analyze the effects of FMT on metabolites and spermatogenesis.

### Heterochronic FMT mitigates the decline of spermatogenesis in old mice

To determine whether and how FMT affects spermatogenesis, we first examined the sperm concentration and motility of all groups of mice following FMT by using computer-assisted semen analysis (CASA). We found that when young mice received microbiota donated from the old mice (o FMT y), their sperm concentration decreased (Fig. 1B–D), as well as some parameters of sperm motility such as curve-line velocity (VCL), amplitude of lateral head displacement (ALH), and straightness (STR) were consistently reduced (Fig. S1A–E). In contrast, old mice that received young donor microbiota (y FMT o) showed increased sperm concentration and motility including progressive motility (grade A + B sperm) and total motility (grade A + B + C sperm) (Fig. 1B–D). Of note, both the o FMT y and y FMT o mice showed no difference in body weight and testis tissues index (testis weight/body weight) when compared with the corresponding control mice (Fig. S1F–H). As for the donor mice, sperm concentration (Fig. S2A), sperm motility including progressive motility and total motility (Fig. S2B–C), and other parameters of sperm motility were also decreased in old mice when compared to young group (Fig. S2D–I).

In addition, H&E staining showed that o FMT y mice had decreased spermatogenic cells and spermatids in the seminiferous tubules, and the seminiferous tubules were also disordered (Fig. 1E,F). Consistently, immunofluorescence staining demonstrated the numbers of DAZL^+^ cells (spermatogonia), SYCP3^+^ cells (spermatocyte), TNP1^+^ cells (spermatid), and PGK2^+^ cells (spermatozoa) in seminiferous tubule were reduced in testis tissues of the o FMT y mice (Fig. 1G,H). On the contrary, y FMT o mice showed rescued spermatogenesis with increased spermatogenic cells (Fig. 1I–L). In addition, for spermatogenesis of the old donor mice, we observed disordered seminiferous tubules and decreased spermatogenic cells (Fig. S2J–K). These findings suggest that the GM plays a significant role in spermatogenesis and that heterochronic FMT can impair or rescue spermatogenesis in mice depending on the age of the donor mice.

### Gut microbiota dysbiosis of the old donor mice

**Fig. 2:**
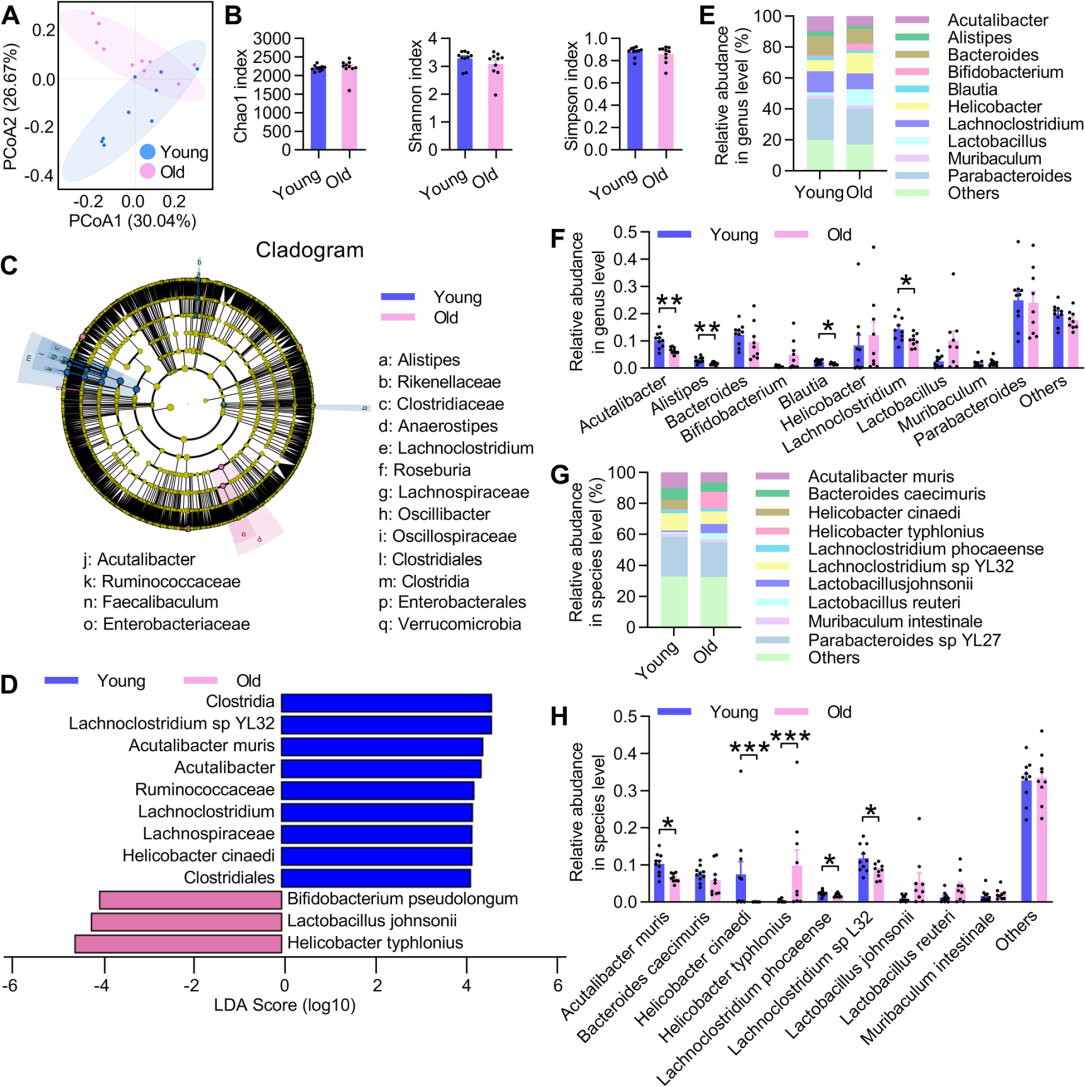
Gut microbiota dysbiosis of old donor mice. **A** Principal coordinate analysis (PCoA) between young and old donor groups. **B** The α-diversity including Chao1, Shannon and Simpson index. **C**,** D** Statistical differences between young and old groups were identified using the line discriminant analysis (LDA) effect size (LEfSe) method. Cladogram illustrating the output of the LEfSe algorithm. Significantly distinct taxonomic nodes are colored, and the branch areas are shaded according to the effect size of the taxa (**C**). Taxa enriched in the young (blue), and old (pink) groups with LDA score ≥ 4 are indicated (**D**). **E** The top ten bacteria with maximum abundance of at the genus level. **F** Significant changes in abundance at the genus level. **G** The top ten bacteria with maximum abundance at the species level. **H** Significant changes in abundance at the species level. All data are presented as mean ± SEM. *P < 0.05; **P < 0.01; ***P < 0.001. Data are analyzed by two-tailed unpaired Student’s* t* test or *Wilcoxon* test. *n* = 10 mice per group

Next, we performed shotgun metagenomic sequencing using cecum feces of young and old mice to evaluate the effects of GM on spermatogenesis. Using principal coordinate analysis (PCoA) based on Bray–Curtis distances, we found that the overall β-diversity of the GM composition was clearly distinct between young and old groups (Fig. 2A). Meanwhile, the α-diversity assessed by the Chao1, Shannon, and Simpson index exhibited no significant difference between the two groups (Fig. 2B). To identify the key phylotypes that were significantly altered in the old group, we used the linear discriminant analysis (LDA) effect size (LEfSe) method to analyze the validated sequences at the genus and species levels. At the genus level, *Lachnoclostridium* and *Acutalibacter* were enriched in the young group, whereas at the species level, *Helicobacter cinaedi*, *Acutalibacter muris*,* Lachnoclostridium sp. YL32*, and* Helicobacter typhlonius*, *Lactobacillus johnsonii*, and* Bifidobacterium pseudolongum* were enriched in the young and old group, respectively (Fig. 2C,D).

The composition of the overall GM was further compared by analyzing the degree of bacterial taxonomic similarity at the genus and species levels. At the genus level, the abundance of *Acutalibacter*, *Alistipes*,* Blautia*, and *Lachnoclostridium* was decreased in the old group (Fig. 2E,F). In addition, our high-quality metagenomic data enabled us to analyze the GM composition at the species level. The identification of the ten most abundant species showed a decreased abundance of *Acutalibacter muris*, *Helicobacter cinaedi*, *Lachnoclostridium phocaeense*, and *Lachnoclostridium sp_L32* and an increased abundance of *Helicobacter typhlonius* in the old group (Fig. 2G,H). Moreover, we presented all the gut microbiota that have a LDA score above 3 (Fig. S3A) and the top 25 species of young and old groups using the log2-fold change (Log2FC) illustration method (Fig. S3B). Taken together, these results reveal that the microbial community structures were different in the GM of young and old mice.

### Gut microbiota-derived 3-HPAA and the related metabolites are changed in heterochronic FMT mice

Given that GM disturbance is closely associated with metabolism of many metabolites, we hypothesized that changes in metabolism of GM may underlie the effects of GM on spermatogenesis. To test this, we performed non-targeted gas chromatography coupled to mass spectrometry (GC–MS) on cecum feces for young and old donor mice. This enabled us to identify 162 metabolites (Data S1), 20 of which were differentially enriched between two groups (Fig. S4). The volcano plots clearly presented the differential microbiotic metabolites in intestine, with 12 beneficial metabolites and 8 detrimental ones (Fig. S4A). Of note, partial least squares discriminant analysis (PLS-DA) showed that the gut metabolites were different between young and old donor mice (Fig. S4B). Interestingly, the intensities of some microbiome-derived metabolites, such as glycolic acid, 3-indolepropionic acid (3-IPA), 3-hydroxyphenylacetic acid (3-HPAA), 2-hydroxyphenylacetic acid (2-HPAA), and oxoadipic acid, were decreased while the intensity of fumaric acid was increased in old mice (Fig. S4C–H). To confirm these findings, we examined the relationship between the differentiated metabolites and the species of GM using *Spearman* correlation analysis and identified 50 correlated species and 20 correlated metabolites (Fig. S5). These data demonstrate that GM indeed affects the production of metabolites in cecum of donor mice.

**Fig. 3:**
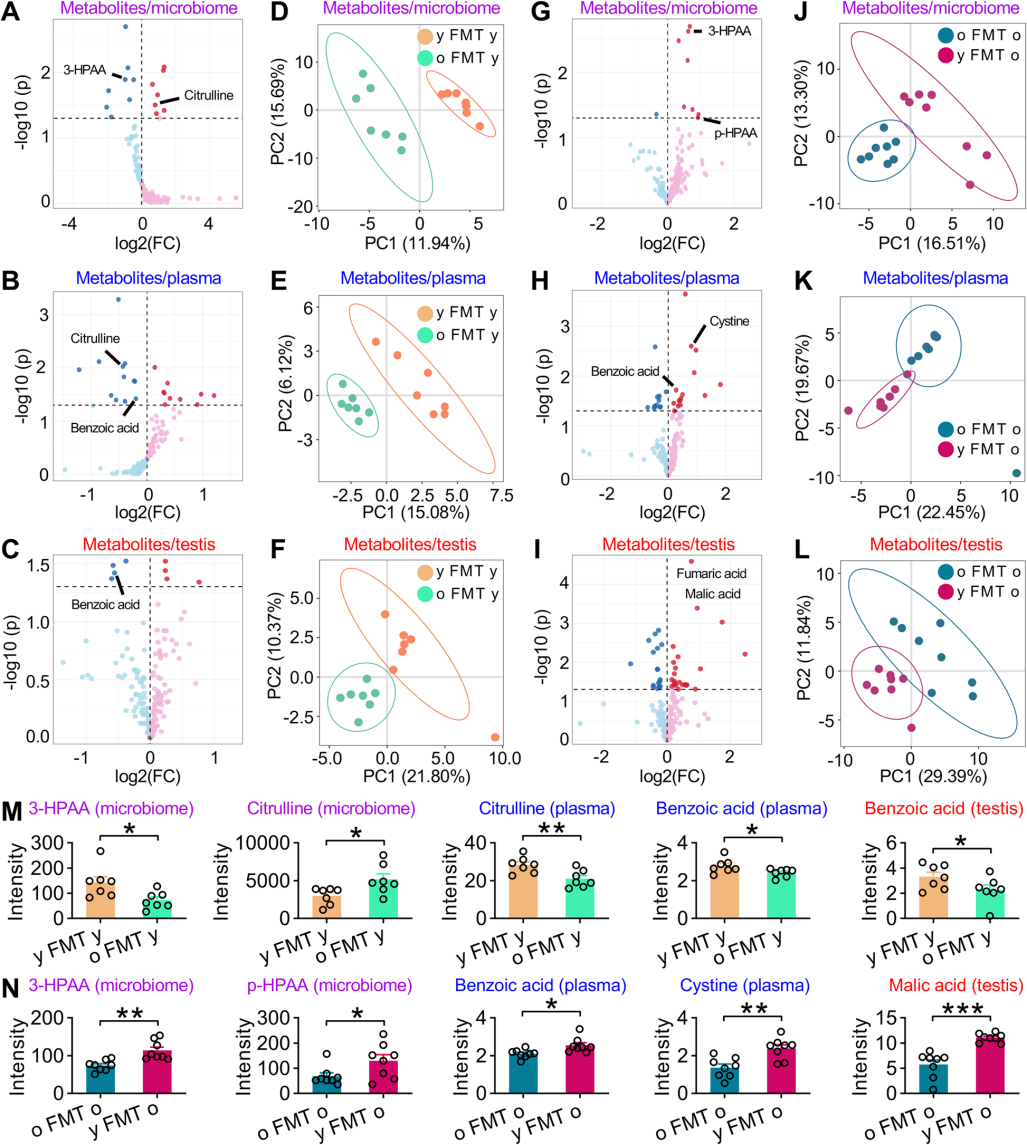
GC–MS metabolomics analysis conducted for microbiome, plasma, and testis tissues of all the FMT groups.** A**–**C** The volcano plots of metabolites for the damaged comparison (o FMT y group and y FMT y) in the microbiome (**A**), plasma (**B**), and testis tissues (**C**). **D**–**F** Partial least squares discriminant analysis (PLS-DA) of metabolites for the damaged comparison in microbiome (**D**), plasma (**E**), and testis tissues (**F**). **G**–**I** The metabolites for rescued comparison (y FMT o and o FMT o) shown as volcano plots in the microbiome (**G**), plasma (**H**), and testis tissues (**I**). **J–L** PLS-DA of metabolites in microbiome (**J**), plasma (**K**), and testis tissues (**L**). Significantly regulated metabolites between groups were determined by absolute FC (fold change) > 1 and *p* value < 0.05 with the representative metabolites indicated (**A**–**C** and **G**–**I**). **M** The intensity of representative metabolites such as 3-Hydroxyphenylacetic acid (3-HPAA) and citrulline in microbiome, citrulline and benzoic acid in plasma, and benzoic acid in testis of y FMT y and o FMT y mice. **N** 3-HPAA and p-HPAA in microbiome, benzoic acid and cystine in plasma, and malic acid in testis of o FMT o and y FMT o mice. All data are presented as mean ± SEM. *P < 0.05; **P < 0.01; ***P < 0.001. Data are analyzed by two-tailed unpaired Student’s* t* test or *Wilcoxon* test. *n* = 7–8 mice per group

To further systemically evaluate influence of GM on host metabolism, we conducted non-targeted metabolome profiling in the cecum feces, plasma, and testis samples of the FMT mice (Data S1). Volcano plots revealed that in the damaged comparison (o FMT y compared to y FMT y), 8/7, 12/10, and 4/4 metabolites were down- and upregulated in the microbiome (Fig. 3A), plasma (Fig. 3B), and testis (Fig. 3C), respectively. PLS-DA showed that the metabolites in the microbiome (Fig. 3D), plasma (Fig. 3E), and testis tissues (Fig. 3F) differed between these two groups. Similarly, in the rescued comparison (y FMT o compared to o FMT o), 1/8, 12/15, and 17/23 metabolites were significantly down- and upregulated in the microbiome (Fig. 3G), plasma (Fig. 3H), and testis (Fig. 3I), respectively, and PLS-DA also revealed the corresponding differences in the metabolites (Fig. 3J–L). The KEGG pathways of these regulated metabolites in testis tissues were shown in Fig. S6. Intriguingly, the levels of 3-HPAA and citrulline in microbiome, citrulline and benzoic acid in plasma, and benzoic acid in testis were reduced in o FMT y mice (Fig. 3M), whereas the intensity of 3-HPAA and p-HPAA in microbiome, benzoic acid, and cystine in plasma, as well as malic acid in testis was increased in y FMT o mice (Fig. 3N). Given that 3-HPAA can be converted to hippuric acid, which can subsequently be metabolized into benzoic acid and malic acid [40], the reduction of 3-HPAA intensity of the gut metabolites of donor old mice and o FMT y mice (Fig. S4E and Fig. 3M) and increased intensity in y FMT o mice (Fig. 3N) suggest that 3-HPAA-related metabolic pathway may be a major contributor to the downregulation of spermatogenesis in old mice and alleviation of impaired spermatogenesis of y FMT o mice.

### The administration of 3-HPAA ameliorates the impact of aging on spermatogenesis

**Fig. 4:**
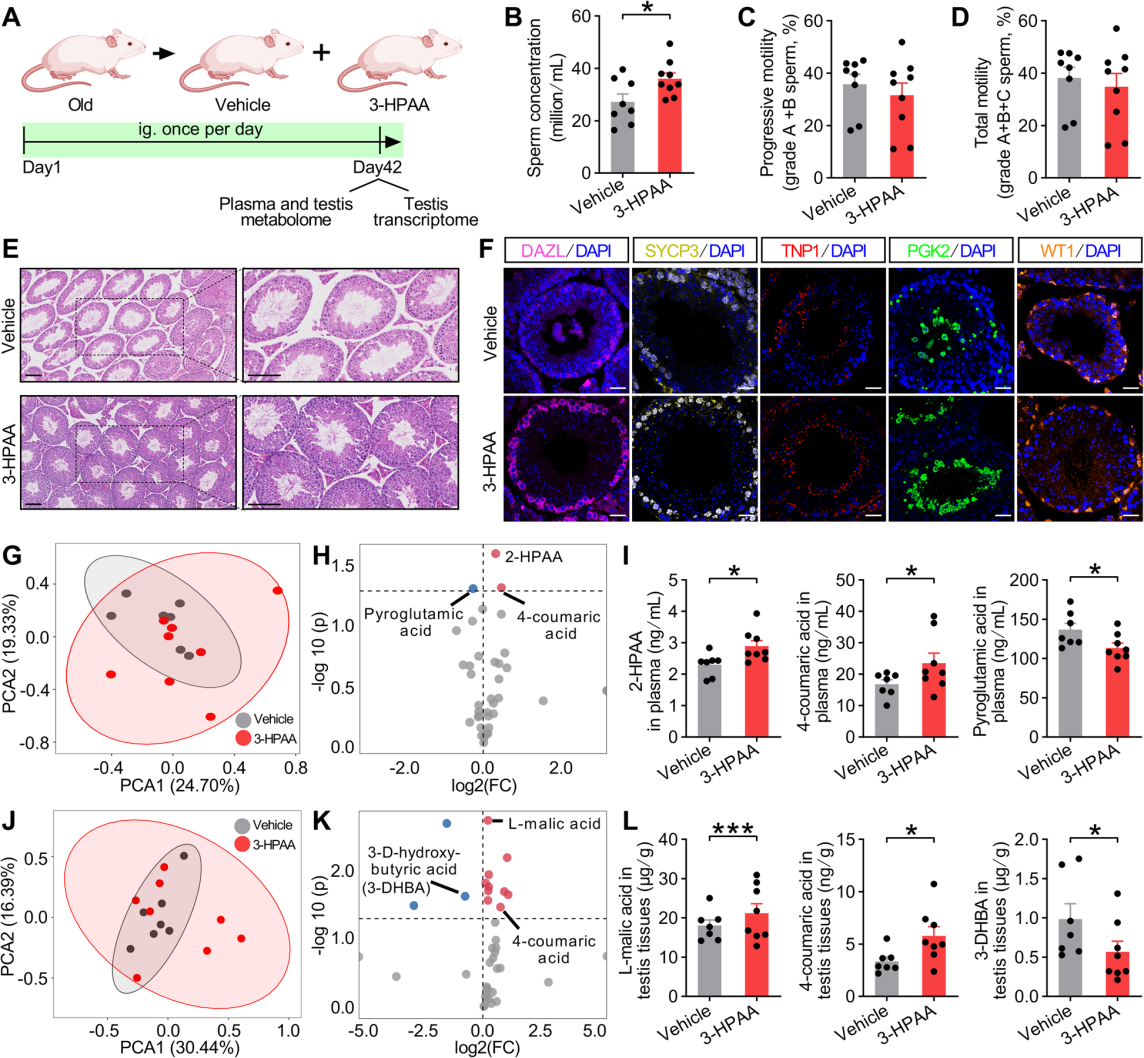
3-HPAA treatment promotes spermatogenesis of old mice through its metabolism pathway. **A** The experimental workflow of 3-HPAA treatment of old mice. Administration of normal saline was used as control. **B**–**D** Sperm concentration (**B**) and sperm motility including grade A + B sperm (**C**) and grade A + B + C sperm (**D**) of old mice with or without 3-HPAA treatment. **E** H&E staining on testis tissues of vehicle and 3-HPAA mice. Scale bar = 100 μm. **F** Representative images of immunofluorescence staining for DAZL (spermatogonia marker), SYCP3 (spermatocyte marker), TNP1 (spermatid marker), PGK2 (spermatozoa marker), and WT1 (Sertoli cell marker) in the testis tissues of old and 3-HPAA mice. Scale bar = 25 μm. **G**–**I **Unsupervised principal component analysis (PCA) (**G**) and the volcano plots (**H**) showed the differentiated metabolites as well as the abundance of 2-HPAA, 4-coumaric acid, and pyroglutamic acid (**I**) in plasma of the two groups. **J**–**L** PCA (**J**) and the volcano plots (**K**) showed the differentiated metabolites as well as the abundance of L-malic acid, 4-coumaric acid, and 3-D-hydroxybutyric acid (3-DHBA) (**L**) in testis of these two groups. Significantly regulated metabolites were determined by absolute FC (fold change) > 1, *p* value < 0.05 and representative metabolites were indicated (**H** and **K**). All data are presented as mean ± SEM. *P < 0.05, ***P < 0.001. Data are analyzed by two-tailed unpaired Student’s *t* test or *Wilcoxon* test. *n* = 7–9 mice per group

To test our hypothesis mentioned above, we investigated whether treatment of 3-HPAA has a therapeutic effect on spermatogenesis in old mice. Old mice were intragastrically administrated with 3-HPAA (3-HPAA mice) or normal saline (vehicle mice) for 6 weeks, then blood and testis tissues were collected at the end of the experiment (Fig. 4A). We found that sperm concentration was increased in 3-HPAA mice although sperm motility was not altered (Fig. 4B–D, Fig. S7A–F). Subsequent H&E and immunofluorescence staining demonstrated that 3-HPAA mice displayed improved spermatogenesis, better organized seminiferous tubules, and increased DAZL^+^, SYCP3^+^, TNP1^+^, and PGK2^+^ cells (Fig. 4E, F). To identify metabolites underpinning the microbiome-dependent alleviation of the disordered spermatogenesis, we performed targeted metabolome in the plasma and testis samples of these two groups (Data S2). PCA showed differentiated dominant metabolites in plasma (Fig. 4G) and the volcano plots clearly showed the 1/2 metabolites were significantly down- and upregulated in the plasma (Fig. 4H) of 3-HPAA mice. Specifically, we found the abundance of 2-HPAA, 4-coumaric acid, and pyroglutamic acid was augmented or diminished in the plasma of 3-HPAA mice (Fig. 4I). Likewise, dominant metabolites also differed in the testis tissues (Fig. 4J) with 3/10 metabolites being significantly down- and upregulated in 3-HPAA mice (Fig. 4K). The abundance of several metabolites such as L-malic acid, 4-coumaric acid was increased, whereas 3-D-hydroxybutyric acid (3-DHBA) was decreased (Fig. 4L). Together, these data prove that microbiome-derived 3-Hydroxyphenylacetic acid-related metabolic pathway can alleviate aging-associated spermatogenic dysfunction in mice.

### Restraint of ferroptosis is involved in spermatogenic recovery of 3-HPAA-treated old mice

**Fig. 5:**
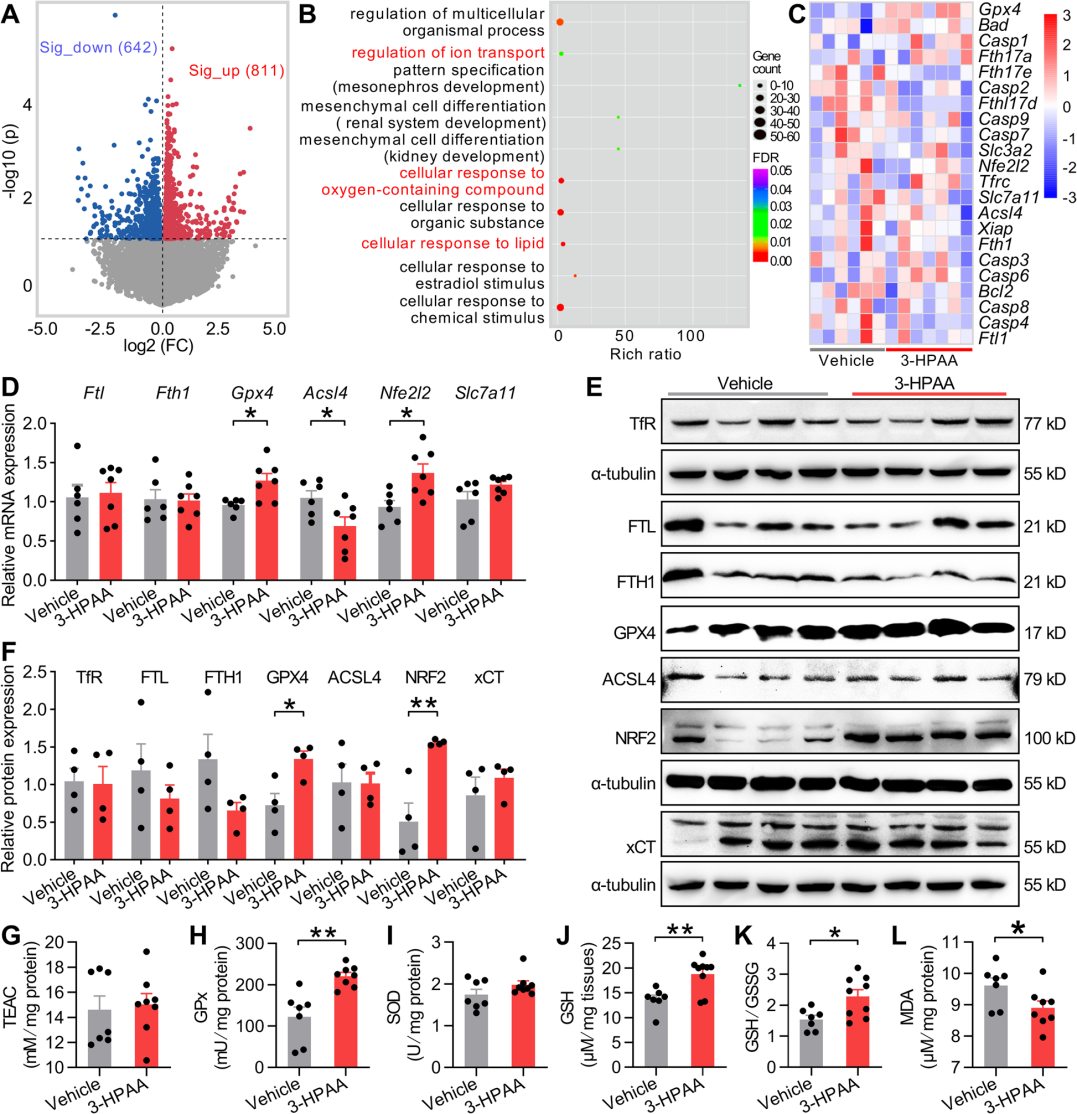
3-HPAA treatment promotes spermatogenesis of old mice through restraint of ferroptosis. **A** The volcano plots of RNA sequencing data showed 642/811 significantly downregulated (blue points) or upregulated (red points) genes in the testis tissues of 3-HPAA mice (*n* = 6–7 mice per group). Significantly regulated genes between groups were determined by absolute FC (fold change) > 1 and *p* value < 0.05. **B** Gene Ontology (GO) analysis of biological process (GO, BP) between 3-HPAA and vehicle mice. The size of bubble indicates gene counts, the color of bubble represents FDR and the important GO categories are highlighted in red. **C** The heat map of genes associated with apoptosis and ferroptosis from RNA sequencing data. Red indicates high abundance and blue indicates low abundance. **D** qRT-PCR validation of mRNA expression of ferroptosis-associated genes including *Ftl*,* Fth1*,* Gpx4*,* Acsl4*, *Nrf2*, and *Slc7a11* in the testis of the two groups and the expression of these mRNA was compared with the housekeeping gene β-actin (*n* = 6–7 mice per group). **E**,** F** Protein expression of TfR, FTL, FTH1, GPX4, ACSL4, NRF2, and xCT in the testis tissues of the two groups (*n* = 4 mice per group). Protein expressions were determined by comparison with α-tubulin on their own gels. **G**–**L** Oxidative stress-related indicators including total antioxidant capacity represented by trolox-equivalent antioxidant capacity (TEAC) (**G**), total glutathione peroxidase (GPx) (**H**), SOD activities (**I**), level of GSH (**J**), ratio of GSH/GSSG (reduced glutathione, GSH; oxidized glutathione disulfide, GSSG) (**K**), and malondialdehyde (MDA) (**L**) (*n* = 7–9 mice per group). All data are presented as mean ± SEM. *P < 0.05; **P < 0.01 and data are analyzed by two-tailed unpaired Student’s *t* test

To further understand the detailed molecular mechanism underlying how 3-HPAA improves spermatogenesis of old mice, we performed RNA sequencing and made comparisons using the testis tissues of vehicle and 3-HPAA mice. 642/811 gene was down- or upregulated in 3-HPAA mice, respectively (Fig. 5A and Data S3), with several Gene Ontology (GO) categories significantly enriched such as regulation of ion transport and cellular response to oxygen-containing compound and lipid (Fig. 5B). Phenols including 3-HPAA and 4-hydroxybenzoic acid (4-HBA) could activate NRF2 [34, 41]. As an important anti-oxidative stress factor, NRF2 regulates the expression of ferroptosis-associated gene such as *Gpx4* and *Slc7a11* [42, 43]. Intriguingly, our transcriptomic results revealed that the expressions of the above genes were altered (Fig. 5C). Additionally, we also discovered that other modulators of ferroptosis, such as *Fth17a/e/d*, *Slc3a2*, *Nfe2l2*, and *Acsl4*, as well as apoptotic genes, including *Bad*, *Casp1/9/7/3/6/8/4*, and *Xiap*, were widely regulated by 3-HPAA treatment (Fig. 5C), suggesting that 3-HPAA may regulate ferroptosis and apoptosis pathways. Consistently, mRNA expression levels of *Gpx4*, *Ascl4*, and *Nrf2* quantified by qRT-PCR were increased in 3-HPAA mice (Fig. 5D). We also conducted Western blot and confirmed that GPX4 and NRF2 proteins were both augmented in the testis tissues of 3-HPAA mice (Fig. 5E,F). To further assess the role of ferroptosis in alleviating spermatogenic dysfunction, protein expression in testis tissues of FMT mice was examined. Indeed, changes of expression in proteins involved in ferroptosis were almost undetected in o FMT y mice (Fig. S8A–H), while the expression of GPX4 and NRF2 was enhanced and ACSL4 was declined (Fig. S8I–P) in y FMT o mice.

As 3-HPAA has been used as a canonical antioxidant, we also assessed the oxidative stress status in the testis tissues of 3-HPAA mice. Expectedly, total glutathione peroxidase (GPx) activity, level of GSH, and ratio of reduced glutathione/oxidized glutathione disulfide (GSH/GSSG) were increased, and an attenuation of malondialdehyde (MDA) abundance was observed (Fig. 5G–L). These results together demonstrate that downregulation of ferroptosis may be involved in spermatogenic recovery of 3-HPAA-treated old mice.

### 3-HPAA-induced therapeutic effects on spermatogenesis of old mice are modulated by GPX4-mediated ferroptosis

**Fig. 6:**
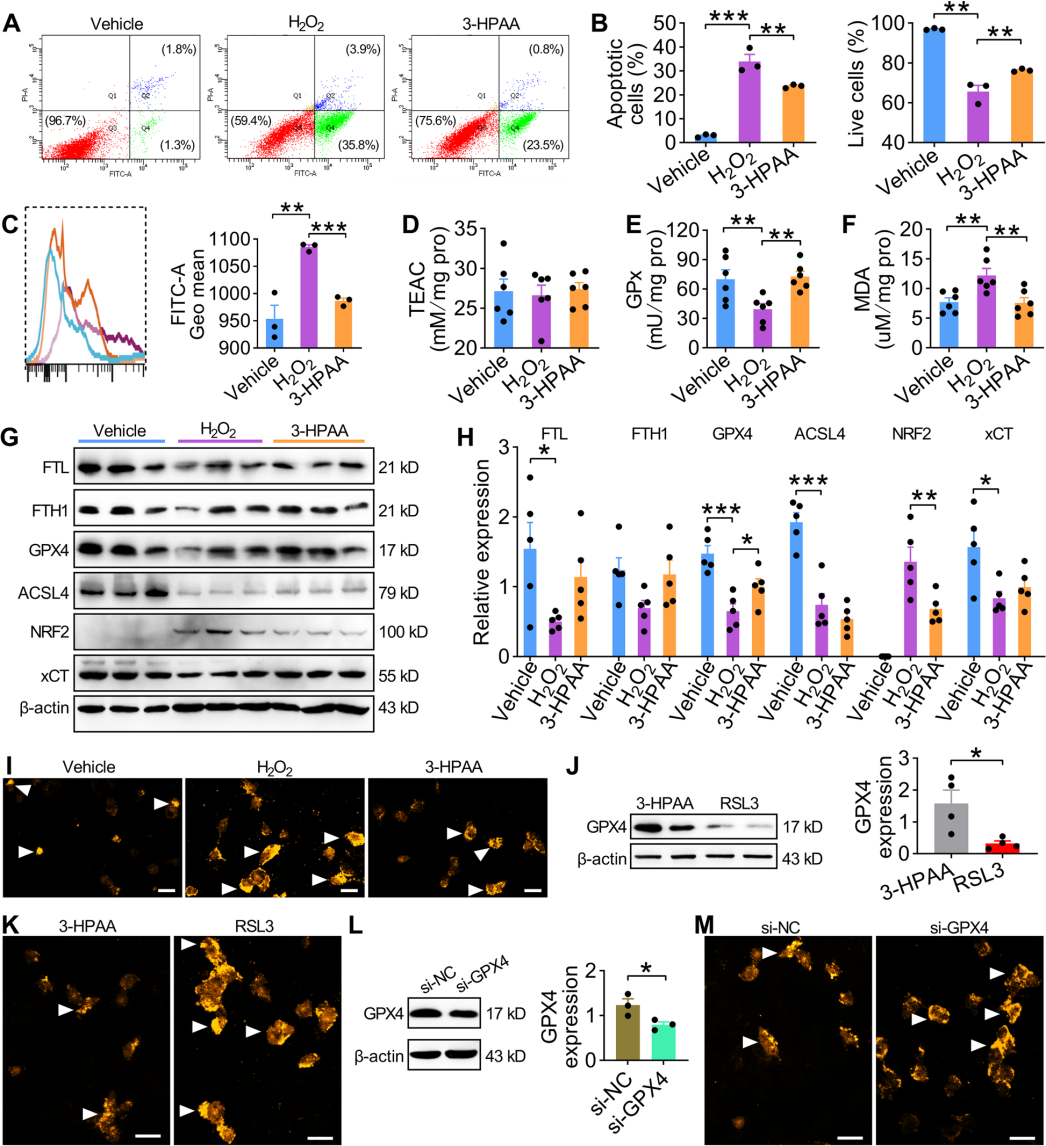
Therapeutic effects of 3-HPAA on spermatogenesis are modulated by GPX-4-mediated ferroptosis.** AB** Typical scatter plots (**A**) and summary (**B**) of Annexin V-FITC/PI assay of GC-2 cells in vehicle, H_2_O_2_, and 3-HPAA groups. GC-2 cells were cultured in normal medium (labeled as vehicle) or treated with 200 μM hydrogen peroxide (H_2_O_2_) for 90 min, and then 50 μM of 3-HPAA (labeled as 3-HPAA) or 0.1% DMSO (labeled as H_2_O_2_) was added into the medium. 48 h later, cells were harvested for subsequent experiments. **C** ROS level of GC-2 cells in three groups. Left: representative image; right: statistics of the ROS level. **D**–**F** Oxidative stress-related indicators including TEAC (trolox-equivalent antioxidant capacity) (**D**), GPx (**E**), and MDA (**F**) in the three groups. **G**, **H** Protein expression of FTL, FTH1, GPX4, ACSL4, NRF2, and xCT in the three groups. **I** Representative images of the iron staining of GC-2 cells after different treatment. Fe^2+^ was detected using FerroOrange assay. **J**,** K** Western blot analysis of GPX4 (**J**) and iron staining (**K**) of GC-2 cells treated with RSL3 (200 nM). **L**,** M** Western blot analysis of GPX4 (**L**) and iron staining of GC-2 cells (**M**) treated with *Gpx4* siRNA. Scale bars = 50 μm. White arrowheads indicated the Fe^2+^ signal-positive cell in **I**, **K**, and **M**. All data are presented as mean ± SEM. For **B**–**H**, *P* values were determined by one-way ANOVA with Sidak’s *post hoc* test. *P < 0.05; **P < 0.01; ***P < 0.001 compared to the vehicle or H_2_O_2_ group. For **J** and **L**, *P* values were determined by two-tailed unpaired Student’s *t* test. *P < 0.05. *n* = 3–6 per group

**Fig. 7:**
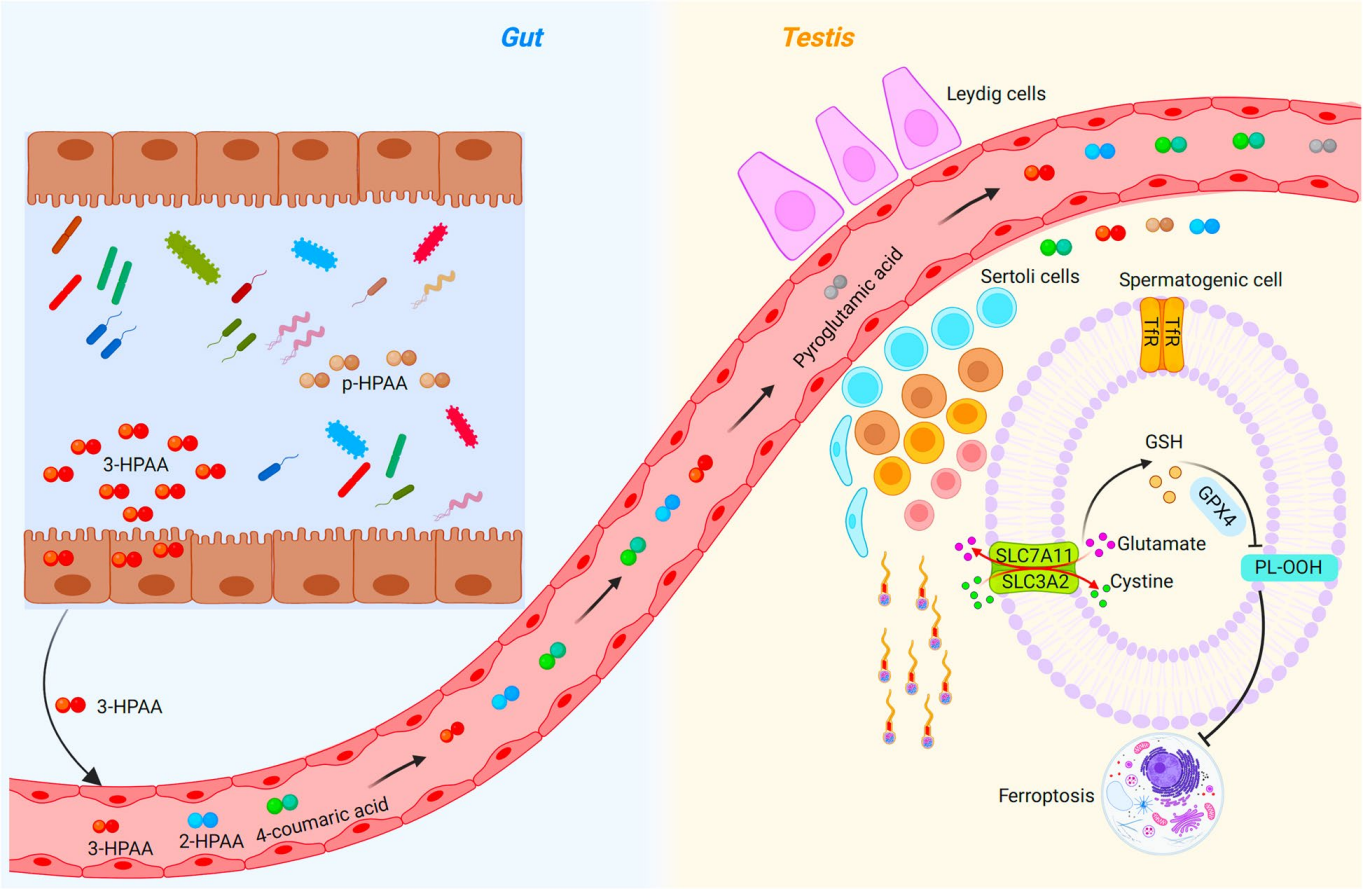
Diagram for the amelioration of gut metabolite 3-Hydroxyphenylacetic acid on the aging-related spermatogenic dysfunction. The altered GM abundance in intestine were closely related to the changed gut bacteria-derived metabolite, 3-Hydroxyphenylacetic acid (3-HPAA). 3-HPAA promotes GPX4-mediated reduction of intracellular phospholipid hydroperoxides (PL-OOH) and 3-HPAA-related metabolic pathway may alleviate the impaired spermatogenesis of aging mice through regulation of ferroptosis

As one of the most important components in restraint of ferroptosis, significantly elevated *Gpx4* RNA expression was detected after 3-HPPA administration (Fig. 5C), suggesting that 3-HPAA may exert its therapeutic effects on spermatogenesis of old mice by regulating GPX4-mediated ferroptosis. To test this hypothesis, we established the senescent GC-2 spd cells by hydrogen peroxide (H_2_O_2_) treatment. Flow cytometry data showed that 3-HPAA treatment alleviated the apoptosis (Fig. 6A,B) and the production of ROS in GC-2 cells (Fig. 6C). Notably, oxidative stress was also decreased after the treatment of 3-HPAA as a higher GPx but lower MDA abundance were observed (Fig. 6D–F). Besides, senescent GC-2 cells treated by 3-HPAA presented a significantly increased GPX4 protein expression, although no change was found for FTL, ACSL4, and xCT (Fig. 6G,H). We also observed that H_2_O_2_ induced Fe^2+^ was decreased after 3-HPAA treatment, suggesting ferroptosis is restrained (Fig. 6I). These results indicate that 3-HPAA may induce ferroptosis restraint by a GPX4-mediated mechanism. Therefore, we stimulated ferroptosis using RSL3 (antagonist for GPX4) or *Gpx4*-siRNA to test this hypothesis. RSL3 treatment significantly decreased the protein expression of GPX4 (Fig. 6J), and the overall abundance of Fe^2+^ was remarkably increased (Fig. 6K), thus partially destructed the alleviation effect of 3-HPAA on ferroptosis. Similarly, siRNA knockdown of GPX4 presented a comparable phenomenon as that of RSL3 (Fig. 6L,M and Fig. S9A–E). These results together demonstrate that the gut metabolite 3-Hydroxyphenylacetic acid may ameliorate the aging-related spermatogenic dysfunction by the alleviation of ferroptosis (Fig. 7).

## Discussion

Dysbiosis of microbiome is closely associated with human aging, and age-related gut microbiota dysbiosis promotes intestinal permeability and progressive decline of health status [44–47]. Disrupted vitamin metabolism induced by dysbiosis of gut microbiota can result in impaired spermatogenesis and sperm motility [48]. In line with these, we observed in the present study that gut microbiota dysbiosis is relevant to the dysfunctional spermatogenesis and decreased sperm motility of old mice, and that the reduction was alleviated by young donor fecal microbiota transplantation to old mice. Furthermore, we identified that the underlying mechanism of this improvement depends on the metabolite 3-HPAA produced by gut microbiota, and 3-HPAA treatment promotes spermatogenesis of old mice through GPX4-induced restraint of ferroptosis.

The dysregulated gut microbiota, which is affected by diet, host genetic, sexual dimorphism, age, mode of birth and antibiotics, is associated with several human diseases including inflammatory bowel diseases, obesity and diabetes, autoimmune diseases, and cardiovascular diseases [49, 50]. For example, *Lactobacillus casei LC122*, *Bifidobacterium longum BL986*, and lifelong dietary omega-3 fatty acid supplementation ameliorates oxidative stress and inflammation in peripheral tissues [51, 52]. Our results revealed that while *Enterobacteriaceae* exhibited enrichment in old mice, *Bacteroidetes* was increased in young mice. Interestingly, these genera displayed analogous patterns in other studies involving human population [53, 54], suggesting that further investigation should be pursued to strengthen the translational relevance of our work. Furthermore, FMT from alginate oligosaccharide dosed mice improves sperm quality and spermatogenesis of busulfan-treated mice [55]. Here, we demonstrated that y FMT o promoted the production of 3-HPAA and its metabolism pathway. 3-HPAA is a major intestinal catabolite of quercetin glycosides and has been shown to possess numerous biological activities, including blood pressure reduction, antioxidant, and anti-apoptosis effects [34, 40, 56, 57]. Our results also revealed that 2-HPAA in plasma as well as 4-coumaric acid in plasma and testis tissues were increased in 3-HPAA-treated mice. Although minor spermatogenetic discrepancies were observed among aged mice from different batches, our results strongly suggest that the mitigation of spermatogenic dysfunction in aging mice could be dependent on the production of the gut microbial metabolite 3-HPAA.

As 3-HPAA has an antioxidant and anti-apoptosis activity, we found that 3-HPAA selectively regulated genes related to several GO categories related to oxygen-containing compound and lipid as well as ferroptosis, a lipid peroxidation-induced cell death that occurs by hallmark mechanisms of iron metabolism disturbance and iron overload [58]. Our results showed that both mRNA and protein expression of GPX4 and NRF2 were augmented after 3-HPAA treatment, indicating the key roles of ferroptosis in 3-HPAA induced alleviation of spermatogenic dysfunction in old mice. Recently, ferroptosis has been shown as a main cause of myocardial infarction, traumatic brain injury, and diabetic nephropathy [58–60]. Competence of ferroptosis, such as inhibition of glutathione defense network and upregulation of p38α-lipid ROS circulation, is also critical for Di (2-ethylhexyl) phthalate (DEHP)-induced blood-testis barrier dysfunction in testis tissues [61, 62]. Similarly, we found that antioxidant elements such as GPx and GSH were upregulated and lipid peroxidation production-MDA was downregulated in the testis tissues of 3-HPAA-treated old mice. Additionally, we observed a significant decrease in ferroptosis after the treatment of 3-HPAA to H_2_O_2_-induced senescent GC-2 cells. Ferroptosis induction or *Gpx4* gene silencing further proved the role of GPX4 in the alleviation effects of 3-HPAA treatment on senescent cells by restraining ferroptosis.

We also note that some limitations exist in our study. For instance, which bacteria species participates in the metabolism of 3-HPAA and the causal relationship of 3-HPAA and ferroptosis is still not clear. Nevertheless, our work provided evidence that a reduction of gut 3-HPAA may result in the spermatogenic dysregulation in old mice. We discovered that o FMT y mice have an impaired spermatogenesis whereas spermatogenesis of y FMT o mice was rescued, which may rely on the gut microbial metabolite 3-HPAA, and 3-HPAA treatment promotes spermatogenesis of old mice through the GPX4-induced restraint of ferroptosis. Overall, our results provide a novel mechanism of dysregulated spermatogenesis of old mice and 3-HPAA may be a potential therapy for fertility decline of aging male in clinical practice.

### Supplementary Information

**Additional file 1: Figure S1.** The sperm motility parameters of all FMT mice. **Figure S2.** Sperm quality and spermatogenesis of young and old donor mice. **Figure S3.** Different microbiota between young and old mice. **Figure S4.** Characterization of metabolites of cecum feces in young and old donor mice. **Figure S5.** Spearman correlation analyses between gut microbiota and differentiated gut metabolites. **Figure S6.** KEGG pathway analysis of the significantly regulated metabolites between groups in the testis tissues of the y FMT o and o FMT o groups. **Figure S7.** Sperm motility parameters of 3-HPAA-treated-old mice. **Figure S8.** Ferroptosis-related protein expression of o FMT y and y FMT o mice. **Figure S9.** Expression and knockdown efficiency of GPX4 siRNA. **Table S1.** PCR primer sequences. **Table S2.** GPX4 siRNA nucleotide sequences.

**Additional file 2: Data S1.** Non-targeted metabolome in the microbiome, plasma, and testis samples of donor or FMT mice.

**Additional file 3: Data S2.** Targeted metabolome in the plasma and testis of the 3-HPAA and vehicle mice.

**Additional file 4: Data S3.** RNA sequencing of the testis tissues of the 3-HPAA and vehicle mice.

## Data Availability

The metagenomic sequencing raw reads generated during this study are freely available at SRA (https://www.ncbi.nlm.nih.gov/sra/) under accession number PRJNA953147. Other data needed to evaluate the conclusions are present in the Supplementary Materials: non-targeted metabolome of the gut, plasma, and testis samples of donor or FMT mice are presented in Additional file Data S1; targeted metabolome of the plasma and testis of the 3-HPAA and vehicle mice are included as Additional file Data S2; RNA sequencing of the testis tissues of the 3-HPAA and vehicle mice are attached in Additional files Data S3.
