# Supplementary material for: The gut metabolite 3-hydroxyphenylacetic acid rejuvenates spermatogenic dysfunction in aged mice through GPX4-mediated ferroptosis
Source: Microbiome. 2023 Sep 27;11:212. doi: 10.1186/s40168-023-01659-y (PMC10523725; doi:10.1186/s40168-023-01659-y)
Supplement: Supplementary file 2 — Additional file 1: Figure S1. The sperm motility parameters of all FMT mice. Figure S2. Sperm quality and spermatogenesis of young and old donor mice. Figure S3. Different microbiota between young and old mice. Figure S4. Characterization of metabolites of cecum feces in young and old donor mice. Figure S5. Spearman correlation analyses between gut microbiota and differentiated gut metabolites. Figure S6. KEGG pathway analysis of the significantly regulated metabolites between groups in the testis tissues of the y FMT o and o FMT o groups. Figure S7. Sperm motility parameters of 3-HPAA-treated-old mice. Figure S8. Ferroptosis-related protein expression of o FMT y and y FMT o mice. Figure S9. Expression and knockdown efficiency of GPX4 siRNA. Table S1. PCR primer sequences. Table S2. GPX4 siRNA nucleotide sequences. [file 40168_2023_1659_MOESM1_ESM.docx]

**The gut metabolite 3-hydroxyphenylacetic acid rejuvenates spermatogenic dysfunction in aged mice through GPX4-mediated ferroptosis**

**Authors: *Zirun Jin^1, 2, 3, 4†^, Yuzhuo Yang^5†^, Yalei Cao^1†^, Qi Wen^6, 7, 8, 9^, Yu Xi^1^, Jianxing Cheng^1^, Qiancheng Zhao^1^, Jiaming Weng^1^, Kai Hong^1, 6^, Hui Jiang^2, 3, 4*^, Jing Hang^6, 7, 8, 9*^, Zhe Zhang^1, 6*^***

**Figures S1–S9 and Figure legends**

**Tables S1–S2**

**
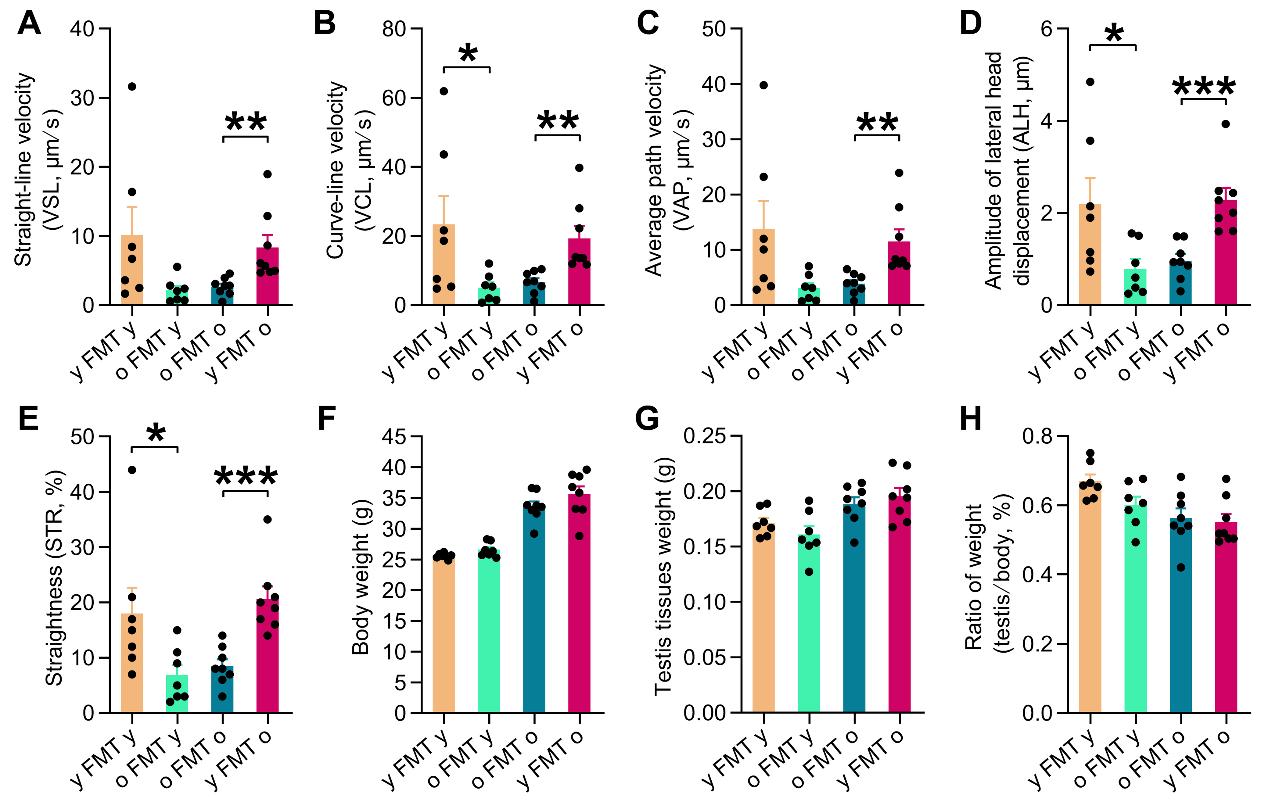
Figure S1. The sperm motility parameters of all FMT mice. (A–E)** Parameters of sperm motility, such as straight-line velocity (VSL) (A), curve-line velocity (VCL) (B), average path velocity (VAP) (C), amplitude of lateral head displacement (ALH) (D), and straightness (STR) (E). **(F–H)** The body weight (F), testis tissue weight (G), and testis index (ratio of testis weight and body weight) (H). All data are presented as mean ± SEM. *P < 0.05; **P < 0.01; ***P < 0.001 and data are analyzed by two-tailed unpaired Student’s *t*-test. n = 7–8 mice per group.

**
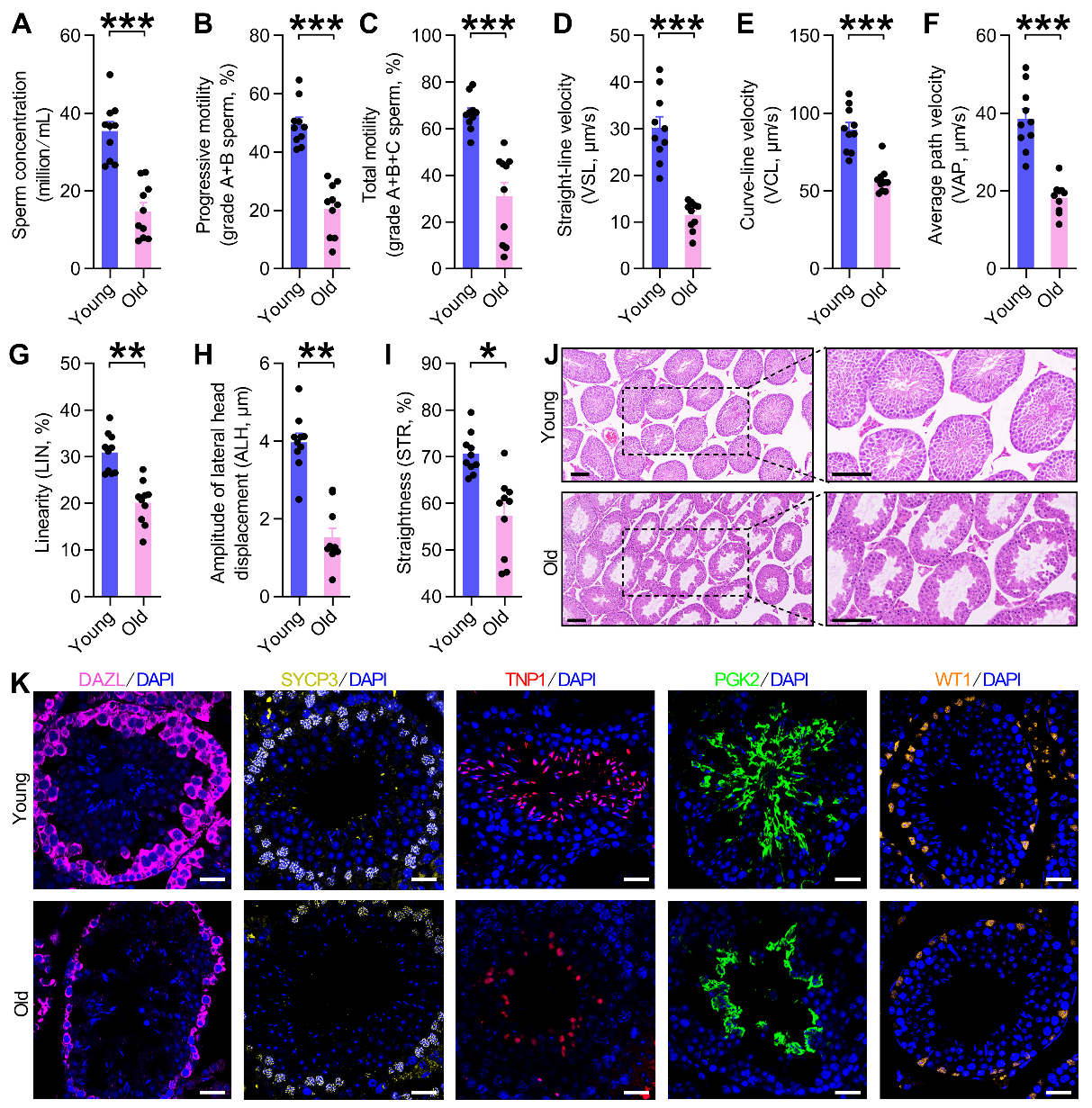
Figure S2. Sperm quality and spermatogenesis of young and old donor mice. (A–I)** Sperm concentration (A) and sperm motility parameters including grade A sperm (B), grade A+B sperm (C), straight-line velocity (VSL) (D), curve-line velocity (VCL) (E), average path velocity (VAP) (F), linearity (LIN) (G), amplitude of lateral head displacement (ALH) (H), and straightness (STR) (I). **(J)** H&E staining of testis tissues in young and old donor mice. Scale bar = 100 μm as indicated. **(K)** Representative images of immunofluorescence staining for DAZL (marker of spermatogonia), SYCP3 (marker of spermatocytes), TNP1 (marker of spermatids), PGK2 (marker of spermatozoa) and WT1 (marker of Sertoli cell) in the testis tissues of young and old donor mice. Scale bar = 25 μm. All data are presented as mean ± SEM. *P < 0.05; **P < 0.01; ***P < 0.001. Data are analyzed by two-tailed unpaired Student’s *t*-test. n = 10 mice per group.

**
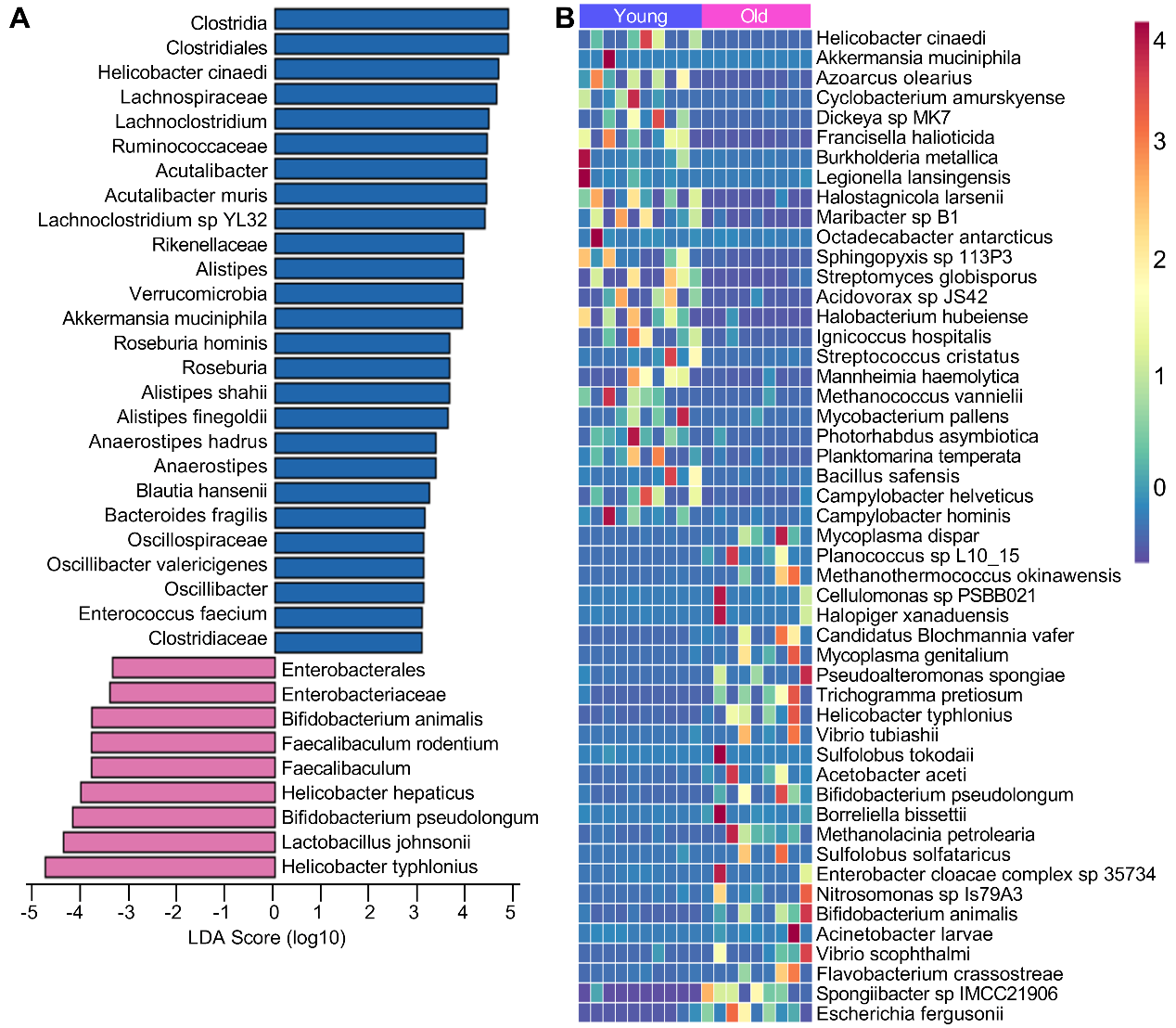
Figure S3. Different microbiota between young and old mice. (A)** All the gut microbiota with a LDA score >= 3. **(B)** The heat map of top 25 differential species in young and old group by using the log2-fold change (Log2FC) illustration method. Blue indicates low abundance and red indicates high abundance.

**
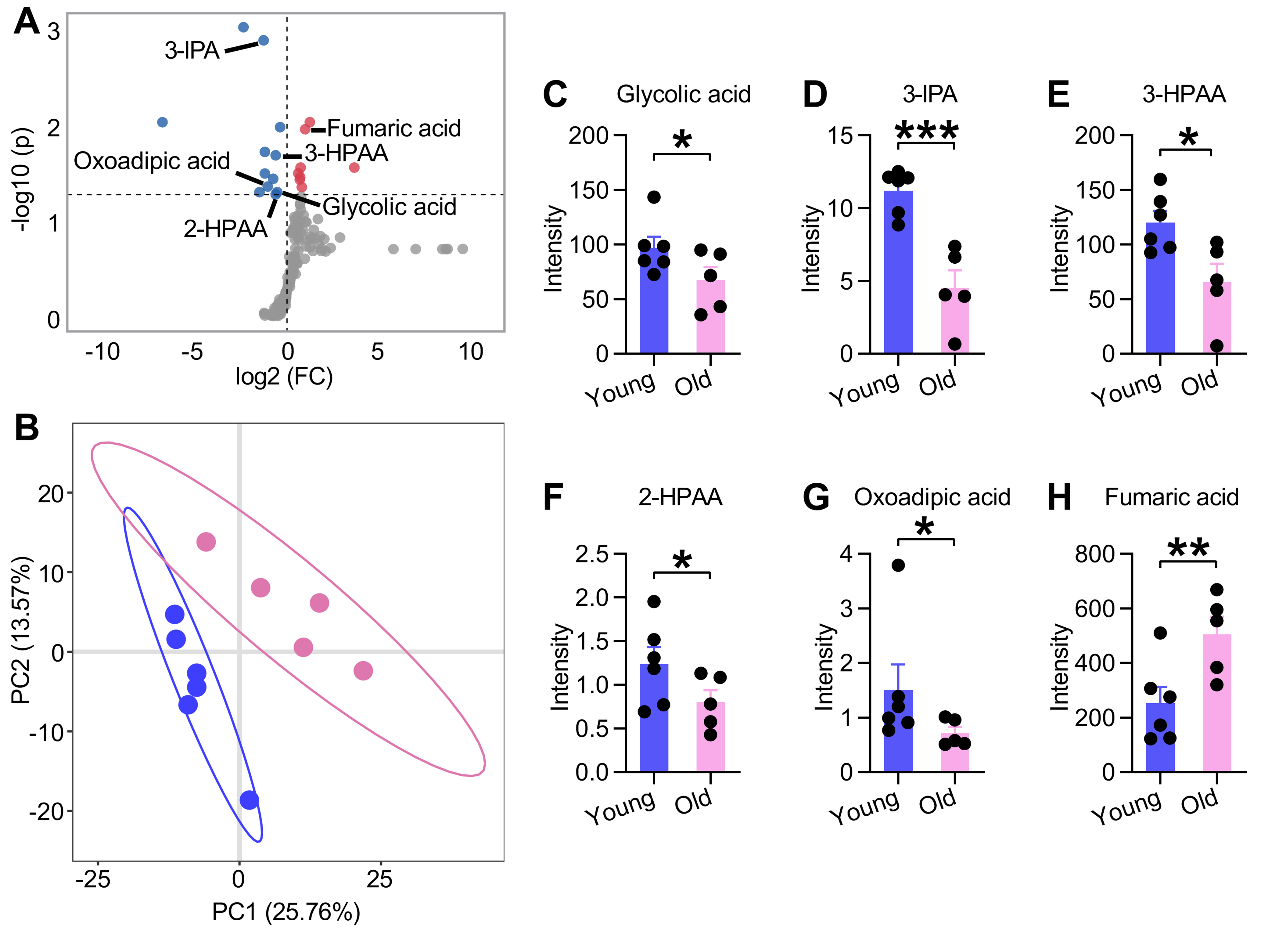
Figure S4. Characterization of metabolites of cecum feces in young and old donor mice. (A)** The volcano plots of microbial metabolites. **(B)** Partial least squares discriminant analysis (PLS-DA) of microbial metabolites. **(C–H)** Intensity of microbiome-derived metabolites including glycolic acid, 3-indolepropionic acid (3-IPA), 3-hydroxyphenylacetic acid (3-HPAA), 2-HPAA, oxoadipic acid and fumaric acid. All data are presented as mean ± SEM. *P < 0.05; **P < 0.01; ***P < 0.001. Data are analyzed by two-tailed unpaired Student’s *t*-test or *Wilcoxon* test. n = 5–6 mice per group.

**
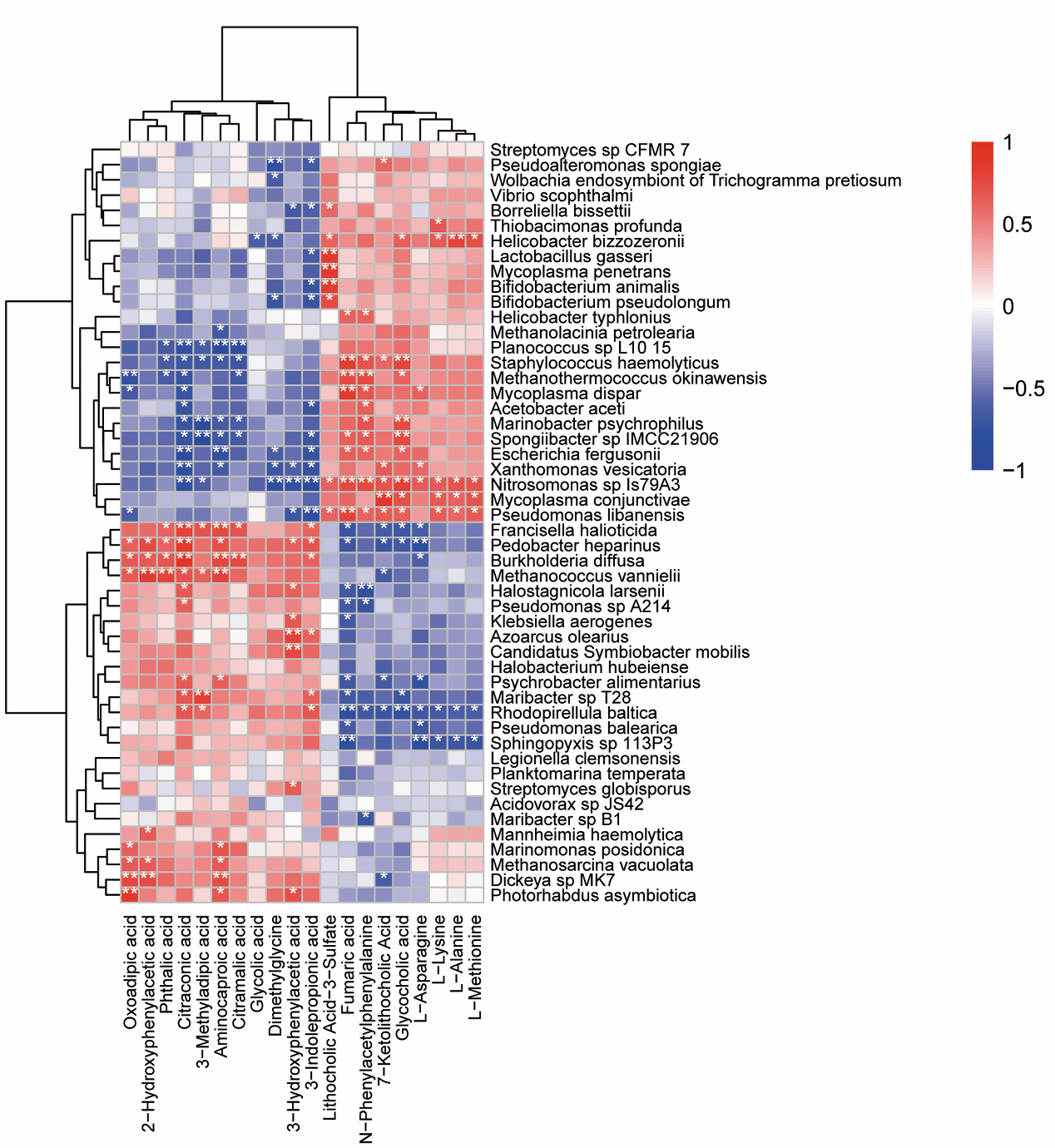
Figure S5. Spearman correlation analyses between gut microbiota and differentiated gut metabolites.** Heat map of the correlation between the GM and metabolites identified 50 correlated species (top 25 in young or old mice) and 20 correlated differentiated metabolites. Blue indicates low abundance and red indicates high abundance.

**
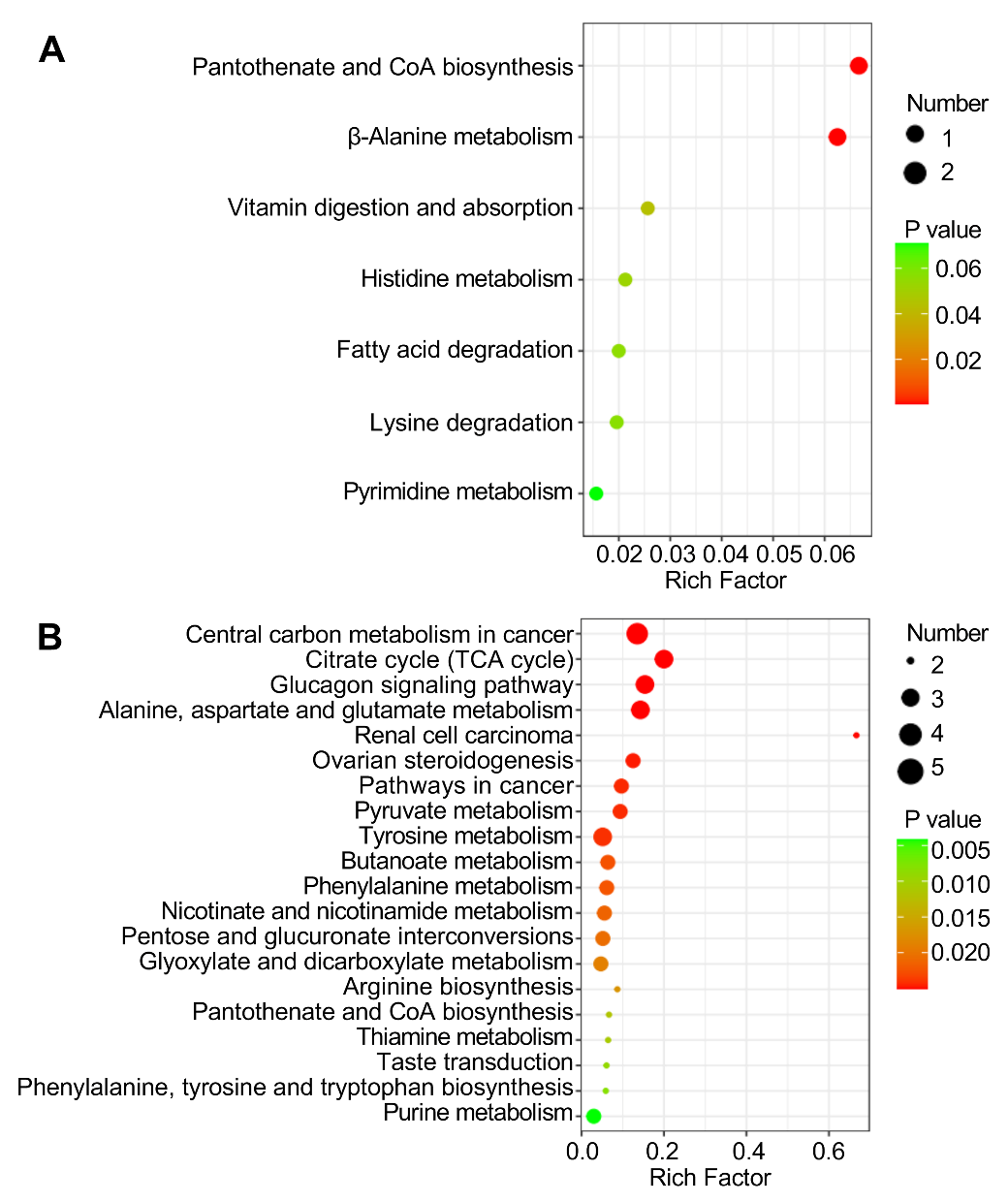
**

**Figure S6. KEGG pathway analysis of the significantly regulated metabolites between groups in the testis tissues of the y FMT o and o FMT o groups.** Kyoto Encyclopedia of Genes and Genomes (KEGG) enrichment analysis of regulated metabolites in the testis tissues of the o FMT y and y FMT y groups (A) as well as the y FMT o and o FMT o groups (B). The size of bubble indicates metabolites counts and the color of bubble represents p value.

**
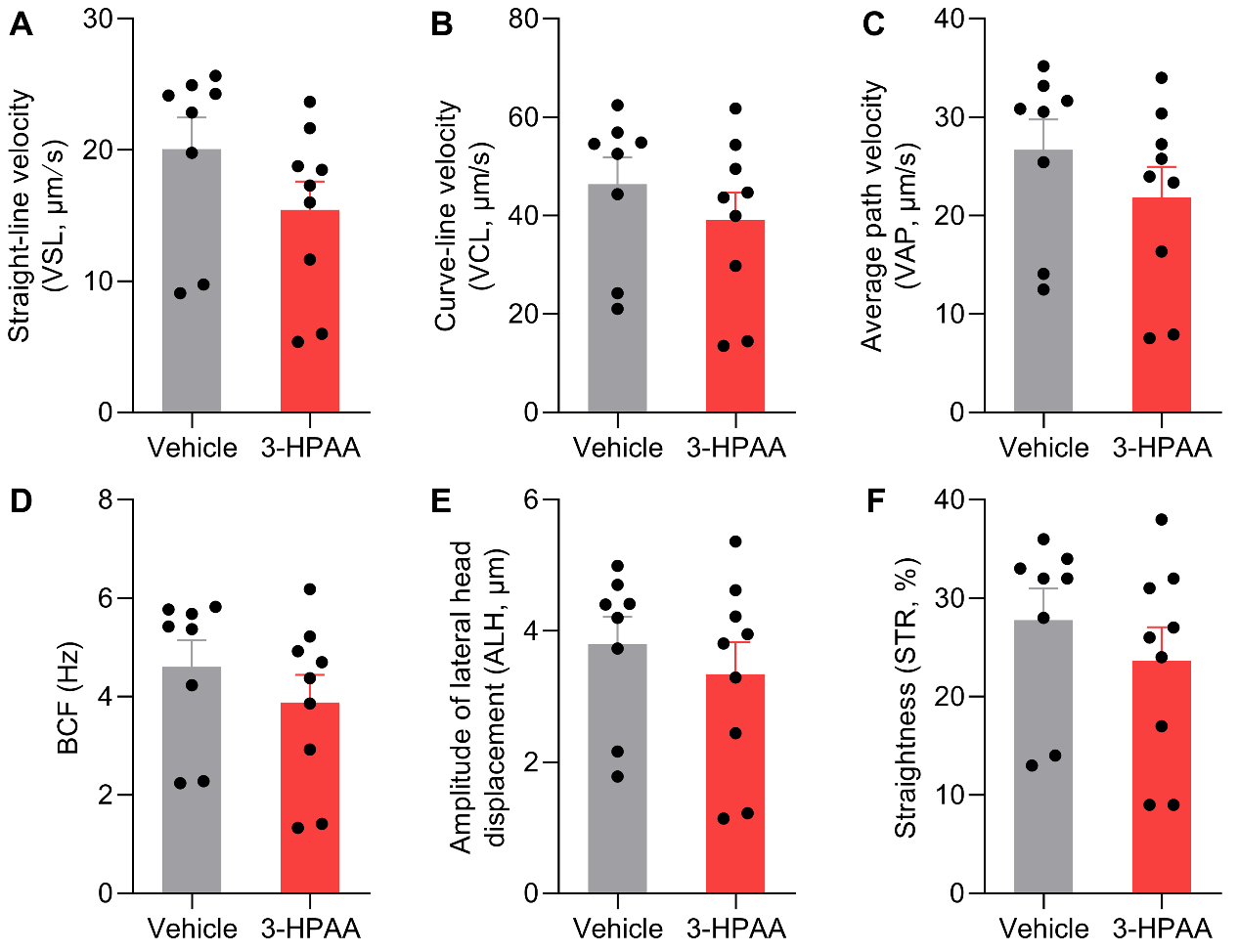
Figure S7. Sperm motility parameters of 3-HPAA-treated-old mice. (A)** Straight-line velocity (VSL). **(B)** Curve-line velocity (VCL). **(C)** Average path velocity (VAP). **(D)** BCF. **(E)** Amplitude of lateral head displacement (ALH). **(F)** Straightness (STR). All data are presented as mean ± SEM. Data are analyzed by two-tailed unpaired Student’s *t*-test. n = 8-9 mice per group.

**
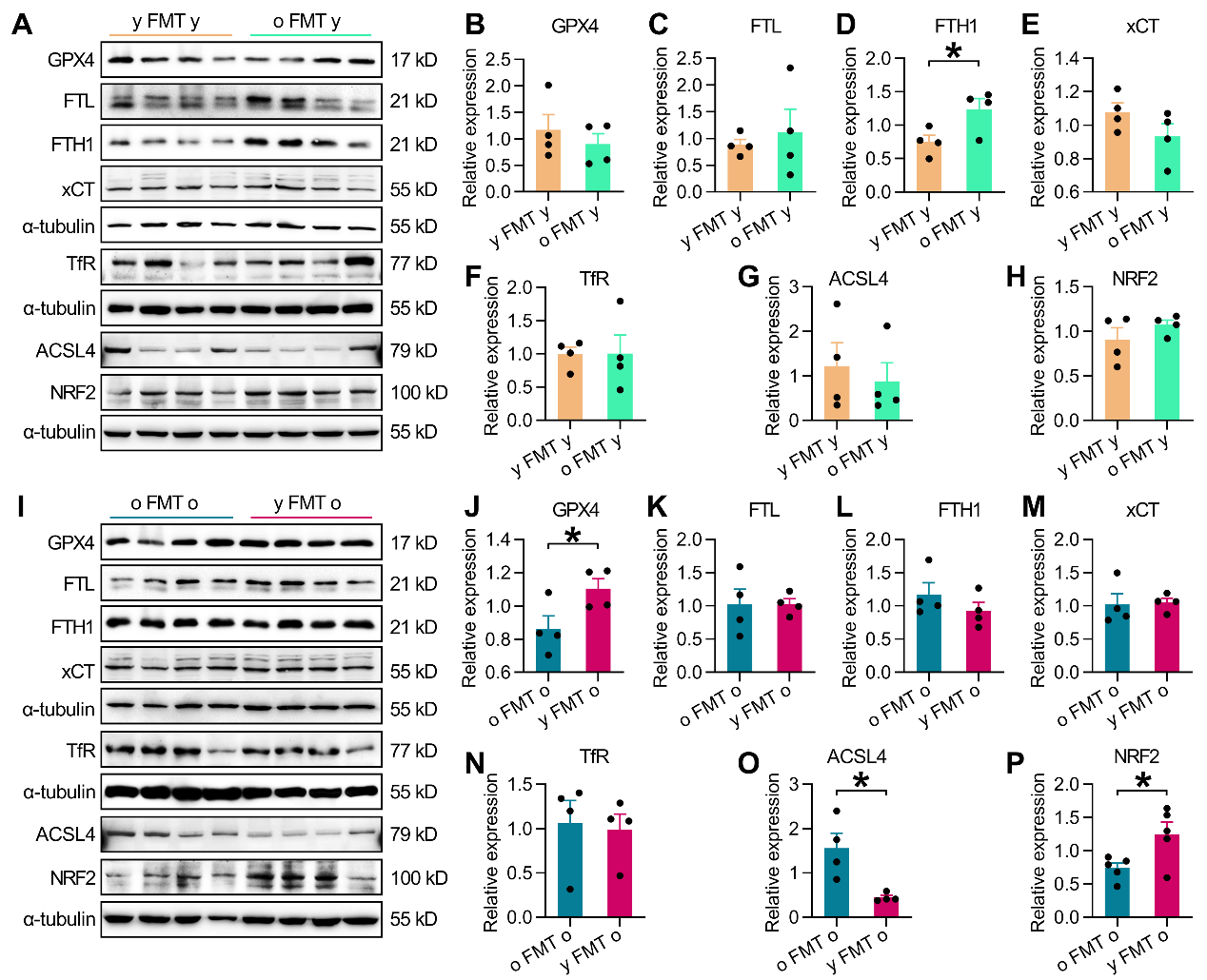
Figure S8. Ferroptosis-related protein expression of o FMT y and y FMT o mice. (A–H)** GPX4, FTL, FTH1, xCT, TfR, ACSL4 and NRF2 protein expression in testis tissues of y FMT y and o FMT y. **(I–P)** GPX4, FTL, FTH1, xCT, TfR, ACSL4 and NRF2 protein expression in testis tissues of o FMT o and y FMT o. Relative expression of proteins was compared with the expression of the housekeeping gene α-tubulin on their own gel. All data are presented as mean ± SEM. *P < 0.05. Data are analyzed by two-tailed unpaired Student’s *t*-test. n = 4 mice per group.

**
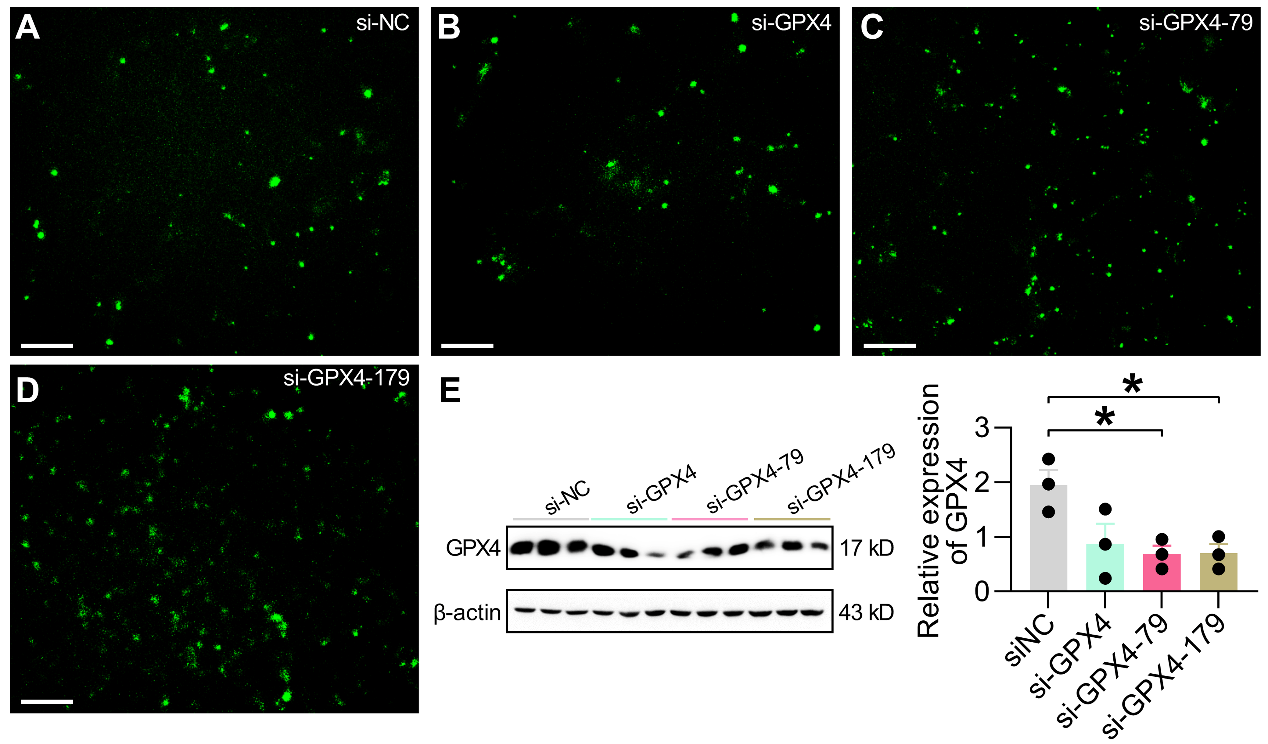
**

**Figure S9. Expression and knockdown efficiency of GPX4 siRNA. (A–D)** Green fluorescence coupled to si-NC, si-*Gpx4*, si-*Gpx4*-79 and si-*Gpx4*-179 validated the transfection efficiency of GC-2 cells. Scale bar = 100 μm. **(E)** GPX4 protein expression was detected by Western blot after siRNA transfection. All data are presented as mean ± SEM. *P < 0.05. Data are analyzed by two-tailed unpaired Student’s *t*-test. n = 3 per group.

**Table S1. PCR primer sequences.**

| Gene (Mouse) | Primer | Sequence |
| --- | --- | --- |
| *Ftl* | Forward (5'-3')  Reverse (5'-3') | CATCTCTGTGACTTCCTGGAAAGCC  GCGGAGGTTGGTCAGATGGTTG |
| *Fth1* | Forward (5'-3')  Reverse (5'-3') | TGCCATCAACCGCCAGATCAAC  ATTCAGCCCGCTCTCCCAGTC |
| *Slc7a11* | Forward (5'-3')  Reverse (5'-3') | CCTCTGACGATGGTGATGCTCTTC  GGTGCTGAATGGGTCCGAGTAAAG |
| *Gpx4* | Forward (5'-3')  Reverse (5'-3') | CATGCCCGATATGCTGAGTGTGG  TAGCACGGCAGGTCCTTCTCTATC |
| *Acsl4* | Forward (5'-3')  Reverse (5'-3') | ATTGGTCAGGGATATGGGCT  AGAGGAGCTCCAACTCTTCCA |
| *Nrf2* | Forward (5'-3')  Reverse (5'-3') | GCATAGAGCAGGACATGGAGCAAG  ACTGATGGCAGCGGAGGAAGG |
| *β-actin* | Forward (5'-3')  Reverse (5'-3') | GGCTGTATTCCCCTCCATCG  CCAGTTGGTAACAATGCCATGT |

**Table S2. *Gpx4* siRNA nucleotide sequences.**

| Name | Primer | Sequence | label |
| --- | --- | --- | --- |
| siRNA | Forward (5'-3')  Reverse (5'-3') | GAUGAAUUAUGUUCAGAAATT  UUUCUGAACAUAAUUCAUCTT | 5'6-FAM  5'6-FAM |
| siRNA-79 | Forward (5'-3')  Reverse (5'-3') | CCGUCUGAGCCGCUUACUUTT  AAGUAAGCGGCUCAGACGGTT | 5'6-FAM  5'6-FAM |
| siRNA-179 | Forward (5'-3')  Reverse (5'-3') | GCGCGCUCCAUGCACGAAUTT  AUUCGUGCAUGGAGCGCGCTT | 5'6-FAM  5'6-FAM |

**Data S1.** Non-targeted metabolome in the microbiome, plasma, and testis samples of donor or FMT mice.

**Data S2.** Targeted metabolome in the plasma and testis of the 3-HPAA and vehicle mice.

**Data S3.** RNA sequencing of the testis tissues of the 3-HPAA and vehicle mice.
